# Circulating tumor DNA precision oncology enables effective and sensitive molecular diagnostics and actionable target detection in pediatric solid tumors - the INFORM experience

**DOI:** 10.1186/s13073-026-01737-4

**Published:** 2026-07-27

**Authors:** Kendra K. Maass, Pitithat Puranachot, Stefanie Volz, Paulina S. Schad, Agnes M.E. Finster, Tom T. Fischer, Barbara C. Jones, Kathrin Schramm, Sophie C. Henneken, Nike Simon, Sophia H. Montigel, Tatjana Wedig, Nathalie Schwarz, Cecilia Zuliani, Petra Fiesel, Christopher Previti, Gnanaprakash Balasubramanian, Florian Iser, Jochen Meyer, Cornelis M. van Tilburg, Till Milde, Olaf Witt, Corinne Rossi, Monika Sparber-Sauer, Stefanie Zimmermann, Thomas Lehrnbecher, Melchior Lauten, Martin Sill, Natalie Jäger, Robert J. Autry, Paul A. Northcott, Felix Sahm, David T.W. Jones, Stefan M. Pfister, Benedikt Brors, Kristian W. Pajtler

**Affiliations:** 1 Department of Pediatric Hematology and Oncology, Faculty of Medicine, Heidelberg University, Heidelberg University Hospital, Heidelberg, Germany; 2 Pediatric Neurooncology , Hopp Children’s Cancer Center Heidelberg (KiTZ), German Cancer Research Center (DKFZ), German Consortium for Translational Cancer Research (DKTK), National Center for Tumor Diseases (NCT), Heidelberg, Germany; 3 Princess Srisavangavadhana Faculty of Medicine, Chulabhorn Royal Academy, Bangkok, Thailand; 4 Applied Bioinformatics, German Cancer Research Center (DKFZ), German Consortium for Translational Cancer Research (DKTK), National Center for Tumor Diseases (NCT), Heidelberg, Germany; 5 Department of Pediatric Hematology and Oncology, Heidelberg University Hospital, Heidelberg, Germany; 6 Pediatric Glioma Research, Hopp Children’s Cancer Center Heidelberg (KiTZ), German Cancer Research Center (DKFZ), German Consortium for Translational Cancer Research (DKTK), National Center for Tumor Diseases (NCT), Heidelberg, Germany; 7 Hopp Children’s Cancer Center Heidelberg (KiTZ), German Cancer Research Center (DKFZ), Heidelberg, Germany; 8 Clinical Cooperation Unit Neuropathology, Hopp Children’s Cancer Center Heidelberg (KiTZ), German Cancer Research Center (DKFZ) German Cancer Consortium (DKTK), Heidelberg, Germany; 9 Core Facility Omics IT and Data Management , German Cancer Research Center (DKFZ), Heidelberg, Germany; 10 Clinical Cooperation Unit Pediatric Oncology, Hopp Children’s Cancer Center Heidelberg (KiTZ), German Cancer Research Center (DKFZ), German Consortium for Translational Cancer Research (DKTK), National Center for Tumor Diseases (NCT), Heidelberg, Germany; 11 Department of Pediatrics and Adolescent Medicine, University Hospital Jena, Friedrich Schiller University Jena, Comprehensive Cancer Center Central Germany (CCCG), Jena, Germany; 12 Center for Pediatric, Adolescent and Women’s Medicine, Pediatric Oncology, Hematology, Immunology , Municipal Hospital of the State Capital Stuttgart (gKAöR), Olgahospital, Stuttgart Cancer Center, Stuttgart, Germany; 13 Department of Pediatric Hematology and Oncology, University Children’s Hospital Tübingen, Tübingen, Germany; 14 Department of Pediatrics, Division of Hematology, Oncology and Hemostaseology, Goethe University Frankfurt, Frankfurt am Main, Germany; 15 Pediatric Hematology and Oncology, University of Lübeck, Lübeck, Germany; 16 Center of Excellence in Neuro-Oncology Sciences (CENOS), St. Jude Children’s Research Hospital, Memphis, USA; 17 Department of Developmental Neurobiology, St. Jude Children’s Research Hospital, Memphis, USA; 18 Department of Neuropathology, Heidelberg University Hospital, Heidelberg, Germany; 19 Faculty of Medicine, Faculty of Biosciences, Heidelberg University, Heidelberg, Germany

**Keywords:** Tumor detection, Therapy monitoring, Pediatric cancer, Orthogonal comparison, Fragment length, Tumor evolution, Tumor heterogeneity, Precision oncology, Tumor board, Clinical implementation

## Abstract

**Background:**

Pediatric solid high-risk malignancies mostly lack established molecular biomarkers for early detection, minimal residual disease assessment, or treatment monitoring. Challenges include small patient numbers, limited sample volumes, low tumor mutational burden, and few recurrent alterations.

**Methods:**

Within the multicenter pediatric precision oncology program INFORM, we prospectively collected liquid biopsies from 130 pediatric patients and optimized cell-free DNA isolation and analysis. Whole-genome, whole-exome, and targeted panel sequencing were performed using liquid biopsy-adapted protocols.

**Results:**

Integrating tissue-derived molecular profiles and orthogonal validation revealed that low-coverage whole-genome sequencing reliably detects circulating tumor DNA. An in silico ctDNA estimation score, combining fragment length and genome segment alterations, improved sensitivity and specificity to 95%, enabling plasma-based tumor detection in 93% of patients. Whole-exome and panel sequencing effectively identified clinically relevant, potentially druggable molecular targets. However, their utility varied substantially across different tumor entities, underscoring the need for entity-specific considerations in the interpretation and application of these methodologies. In-depth analyses demonstrated liquid biopsy’s potential to track tumor evolution, identifying common tumor ancestors and refining patient stratification.

**Conclusions:**

This study advances liquid biopsy methodologies in pediatric oncology and provides a rationale that, as SNVs are more sensitively captured by panel sequencing and WES, while CNVs are better represented by lcWGS and WES. The underlying tumor genomic profile should guide the selection of liquid biopsy assays to optimize clinical decision-making. Systematic liquid biopsy analyses within the pediatric precision oncology INFORM registry enabled a real-world, multicenter comparison of sequencing approaches across high-risk malignancies. By optimizing preanalytical and bioinformatic tools for pediatric settings, we improved plasma-based cancer detection, molecular tumor characterization, and identification of targetable alterations, laying the groundwork for integration into personalized medicine programs and clinical trials.

**Supplementary Information:**

The online version contains supplementary material available at 10.1186/s13073-026-01737-4.

## Background

Children and adolescents with relapsed, progressive, or refractory high-risk malignancies face poor prognoses, prompting the establishment of pediatric precision oncology programs [1], including pediatric MATCH [2], MOSCATO-01 [3], MAPPYACTS [4], the ZERO Childhood Cancer Program [5], iTHER, SM-PAEDS, PROFYLE, and the INFORM (INdividualized Therapy FOr Relapsed Malignancies in Childhood) registry [6–8]. These programs apply comprehensive molecular profiling of tumor tissue to identify the mechanism-of-action-based treatments. Within INFORM, we validated a predefined molecular target prioritization scheme in a prospective, real-world, multicenter setting [7]. The seven-scale algorithm considers the alteration type and its disease-specific relevance and enables the identification of patient subgroups that clinically benefit from matched targeted treatments by demonstrating extended progression-free survival (PFS) in patients receiving matched targeted treatment with high-evidence targets [7, 8].

Unlike leukemias, solid pediatric malignancies often lack molecular biomarkers, such as translocations, for continuous disease monitoring. Reliance on radiological imaging delays therapeutic interventions, limiting opportunities for treatment adaptation. To complement tumor analyses, INFORM explored plasma-based liquid biopsies to identify pediatric cancer biomarkers. Liquid biopsies provide a minimally invasive method for real-time tumor monitoring, capturing spatial and temporal genomic changes [9]. While liquid biopsies are increasingly utilized in adult oncology and first FDA-approved diagnostic tests have become available, their implementation in pediatric oncology is challenging due to small patient cohorts, limited sample volumes, and low somatic mutation frequencies [10–15]. The MAPPYACTS trial applied cell-free DNA (cfDNA) whole-exome sequencing (WES) in pediatric solid tumors but achieved a technical success rate in only one-fourth of cases [4]. Additional studies have demonstrated feasibility in specific tumor types but lacked a comprehensive pan-cancer approach [16, 17]. Recent work in pediatric tumors has shown that cfDNA sequencing can reveal relapse-driving mutations in epigenetic regulators alongside pronounced intratumor heterogeneity and cell-state–specific chromatin accessibility patterns, underscoring its power to uncover both genetic and epigenetic drivers of aggressive disease [18]. We previously established optimized preanalytical protocols for preserving, isolating, and processing cfDNA across multinational, multi-institutional settings, ensuring reliable genomic analyses with minimal sample input [19]. Our prior work also demonstrated that circulating tumor DNA (ctDNA) serves as a prognostic biomarker for minimal residual disease (MRD) in medulloblastoma [20].

To advance liquid biopsy applications in pediatric precision medicine, we implemented a multiplatform sequencing strategy, including whole-genome sequencing (WGS), WES, and targeted panel sequencing, in 130 pediatric patients enrolled in INFORM. Protocols were specifically adapted for small plasma volumes and low cfDNA concentrations. We assessed copy number variations (CNVs), small insertions and deletions (indels), and single-nucleotide variants (SNVs) to enable cancer detection, molecular characterization, and identification of potentially actionable alterations through a tumor type–agnostic approach. To enhance tumor detection sensitivity and specificity, we developed an in silico ctDNA estimation score (CES), which was successfully applied across the entire cohort. Liquid biopsies collected during therapy facilitated disease monitoring and enabled early detection of tumor progression, preceding radiological assessments. Notably, direct comparisons suggest that liquid biopsy can provide complementary insights into spatial tumor heterogeneity beyond single-site tumor biopsies. In our cohort, cfDNA analyses identified alterations consistent with subclonal populations potentially originating from distinct tumor regions or metastatic sites, as well as critical prognostic markers not detected in the available tumor tissue samples. These findings highlight liquid biopsies as a powerful tool for understanding tumor evolution under treatment pressure, offering novel opportunities for molecularly informed patient stratification and therapy adaptation in pediatric oncology.

## Methods

### Study design, eligibility and participants

The INFORM registry is a prospective, noninterventional, multicenter, multinational, feasibility registry that collects clinical, functional (biological), and molecular data. After a pilot phase [6], the registry opened on January 21st, 2015. The study was prospectively registered at the German Clinical Trial Register with the number DRKS00007623. Ethics committee approval for conducting the study, the use of consent forms and scientific evaluation of the data were obtained from Heidelberg University’s review board (S-502/2013; S-795/2020). Eligible patients included children, adolescents and young adults with refractory, relapsed or progressive oncological disease after initial treatment according to the German Society for Pediatric Oncology and Hematology (GPOH) protocol, as well as specific primary indications. The detailed inclusion criteria are outlined in Worst et al. 2016 [6]. The tumor samples and matched germline controls from each patient were subjected to WES, low-coverage whole-genome sequencing (lcWGS), RNA sequencing (tumor only) and methylation array (450 K, 850 K or EPIC array, tumor only) as previously described [6]. All patients whose plasma samples were available (01/2015-08/2019) were included in this study. Blood samples were collected via standard venipuncture, processed at the respective treatment center and shipped together with tumor and germline material. Blood samples were collected before and after surgery. All the plasma samples were stored at -80 °C until further processing. For healthy controls, peripheral blood samples were collected from 10 healthy individuals aged between 24 and 48 years by standard venipuncture in EDTA tubes and centrifuged at 4 °C for 10 min at 1900 × g. In addition, the cohort of healthy controls was expanded by including 22 age-matched pediatric samples EGAD00001007080 [21].

### Study cohort and sample analyses

This study included all patients enrolled in the INFORM registry between January 2015 and August 2019 for whom plasma samples were available. The study cohort comprised 130 pediatric patients, including 61 patients with brain tumors, 55 with sarcomas, and 14 with other solid malignancies. Matched tumor tissue and plasma-derived cfDNA samples were subjected to molecular profiling. Tumor analyses comprised tumor-normal WES, tumor-normal lcWGS, and tumor methylation array profiling. Plasma-derived cfDNA samples were analyzed by lcWGS, WES, and targeted panel sequencing according to sample availability and quality. Following quality control, the final dataset comprised 132 lcWGS analyses, 66 WES analyses, and 74 targeted panel sequencing analyses.

### cfDNA isolation from plasma samples

Supernatants from preprocessed plasma samples were thawed on ice, and cfDNA was isolated via the NucleoSnap cfDNA Kit (Machery Nagel, Düren, Germany) according to the manufacturer’s manual. Elution was performed with 50 µl of nuclease-free water (Thermo Fisher Scientific, Waltham MA, USA). The extracted cfDNA was quantified with a Qubit dsDNA HS Assay Kit (Thermo Fisher Scientific, Waltham, MA, USA), and the fragment size distribution was assessed with a Bioanalyzer High Sensitivity DNA Kit (Agilent, Santa Clara, CA, USA). All cfDNA samples were stored at − 80 °C until further analysis.

### Low-coverage whole-genome sequencing of cfDNA

WGS libraries were prepared with an Accel-NGS 2 S Hyb DNA Library Kit (Swift Biosciences, Ann Arbor, MI, USA) according to the manufacturer’s protocol. cfDNA was used without fragmentation, and when available, the input was adjusted to 100 pg per library. Libraries were ligated with single-indexing adapters and unique molecular identifiers (UMIs; Swift Biosciences, Ann Arbor, MI, USA). The number of amplification cycles was adjusted from 12 to 16 cycles depending on the input yield. No-template and water controls were included in each experiment. Libraries were quantified with a Qubit dsDNA HS Assay Kit (Thermo Fisher Scientific, Waltham, MA, USA), and the fragment size distribution was assessed with a Bioanalyzer High Sensitivity DNA Kit (Agilent, Santa Clara, CA, USA). Libraries were multiplexed in equimolar amounts and sequenced at 100 bp paired ends on a NovaSeq 6000 S4 flow cell (Illumina, San Diego, CA, USA) targeted for 3x mean depth of coverage.

### Whole-exome sequencing of cfDNA

WES libraries were prepared with the Accel-NGS 2 S Hyb DNA Library Kit (Swift Biosciences, Ann Arbor, MI, USA) according to the manufacturer’s recommendations for SureSelect protocols. In brief, truncated adapters of the XT Compatibility Module (Swift Biosciences, Ann Arbor, MI, USA) were used in the ligation I step. The SureSelectXT Target Enrichment System for Illumina Paired-End Multiplexed Sequencing Library protocol was used to capture SureSelectXT Human All Exon, V7 (Agilent, Santa Clara, CA, USA). SureSelectXT libraries were labeled with uniquely indexed adapters in the posthybridization capture amplification step with primers from Agilent. Library quality was again assessed with a Qubit dsDNA HS Assay Kit (Thermo Fisher Scientific, Waltham, MA, USA) and TapeStation (Agilent, Santa Clara, CA, USA). Libraries were pooled in equimolar amounts and sequenced on a HiSeq 4000 paired-end 100 bp (Illumina, San Diego, CA, USA), with 300x mean depth of coverage.

### Panel sequencing of cfDNA

Capture-based panel sequencing was performed via a custom brain tumor panel (Agilent Technologies, Santa Clara, CA, USA) covering the entire coding and selected intronic and promoter regions of 130 genes (0.9 Mb) of particular relevance in CNS tumors [22]. cfDNA was used without fragmentation, and the input was adjusted to 4 ng per library. Libraries were ligated with single-indexing adapters and UMIs (Swift Biosciences, Ann Arbor, MI, USA). Depending on the library yield, 12–16 amplification cycles were performed. Libraries were quantified via the Qubit dsDNA HS Assay Kit (Thermo Fisher Scientific, Waltham, MA, USA), and the fragment size distribution was assessed via the Bioanalyzer High Sensitivity DNA Kit (Agilent, Santa Clara, CA, USA). Libraries were pooled in equimolar amounts and sequenced at 100 bp paired-end on a HiSeq 4000 platform (Illumina, San Diego, CA, USA) targeted for 10,000x mean depth of coverage.

### CNV analysis of low-coverage whole-genome sequencing data

All next-generation sequencing (NGS) data were transferred to the German Cancer Research Center (DKFZ) Omics IT and Data Management Core Facility (ODCF). In-house bioinformatics workflows for sequence alignment and somatic variant calling were performed through the One Touch Pipeline (OTP) service [23]. Briefly, sequencing reads were aligned to the human reference genome GRCh37 (hg19) [24] plus PhiX contig via BWA-MEM (v0.7.15) [25]. Sambamba (v0.6.5) [26] was used for sorting, and the alignment results were indexed. UMI processing was performed via fgbio (v1.1.0; Fulcrum Genomics) and the Picard toolkit to collapse BAM files and identify high-quality, deduplicated molecular consensus reads that were subjected to a second round of sequence alignment with BWA-MEM. The markduplication process was not performed because of the use of UMIs. Picard CollectWgsMetrics and CollectInsertSizeMetrics (v2.21.8) [27] estimated the sequencing quality matrix, including average depth of sequencing coverage and distribution of template size. The CNV calling, segmentation and tumor fraction estimation tool ichorCNA (v0.3.2) [28] was used to calculate normalized log2 ratios from read count information for each 1 MB genomic window. The Panel-of-Normal (PoN) function was used to control for the nature of cfDNA by including healthy control cfDNA samples. Integrating PoN reduced noise and corrected systematic biases arising from different sequencing platforms, sample preparation protocols, and cfDNA-specific genomic features. We implemented a protocol-specific PoN for the Accel-NGS 2S Hyb DNA Library Kit (Swift Biosciences, Ann Arbor, MI, USA). To create a PoN for samples from the Accel-NGS library, we selected UMI-deduplicated BAM files with a mean depth of coverage between 0.24 and 2.35 and implemented the function “diagnose control group” from the R package NIPTeR [29] to create a NIPTControlGroup where samples with z-transformed chromosomal coverage stay in the range [-3,3]. This process involves screening cfDNA samples with high tumor content in cfDNA with large copy number alterations. Since the majority of cfDNA samples contained low amounts of tumor-derived cfDNA, the ichorCNA default settings were modified to improve parameter estimation. The parameters were changed as follows: –ploidy ”c(2,3)” –normal ”c(0.8,0.9,0.95,0.99,0.995)” –maxCN 4 –includeHOMD FALSE –estimateScPrevalence FALSE –scStates ”c()” –chrTrain ”c(1:22)”. LcWGS data with a mean depth of coverage below 0.1 were excluded from the analysis. To assess potential batch effects arising from decentralized sample collection and processing, t-distributed stochastic neighbor embedding (t-SNE) was performed using the Rtsne package on ichorCNA-corrected 1 Mb bin-wise log2 ratio profiles and annotated by sequencing center. To facilitate comparisons between tumor and cfDNA CNVs, reported CNV events were adjusted according to the reported tumor ploidy. IchorCNA detected CNVs on the basis of sequencing coverage segmentation per 1 MB of genomic nonoverlapping windows. On the basis of the segmentation result, the software detected copy number aberrations by fitting a model with a range of parameters for tumor fractions (ichorTF) and tumor ploidy. The CNV profile with the highest likelihood score was considered. Visual inspection of CNV profiles was performed in predefined scenarios where automated segmentation approached its detection limits, such as low tumor fraction, increased noise, or fragmented segments. To ensure reproducibility, manual CNV calls were restricted to clinically relevant loci and required concordant evidence across multiple adjacent bins. Concurrent tumor and cfDNA CNVs were called when the overlapping cfDNA segment reported the same CNV event (amplification or deletion, regardless of reported copy number) as the tumor. Moreover, the overlapping cfDNA CNV had to be covered by more than 20% of the tumor segment.

### Genome-wide methylation array data for CNV and methylation analysis

Genome-wide DNA methylation data from tumor biopsies were generated as part of the standard INFORM pipeline via the Infinium HumanMethylation450 or MethylationEPIC BeadChip (Illumina) [30]. The Conumee package (Bioconductor) was used to infer genome-wide CNVs [31]. We utilized the ‘noob’ algorithm from the minfi package [32] in R for robust preprocessing of methylation data obtained from tumor and liquid biopsy samples. For our analysis, we specifically examined CpGs within the *H19* locus, which were selected on the basis of a study by Coorens *et al*. [33]. To visualize the methylation distribution across samples, we employed the ‘densityPlot’ function available in the minfi package [32].

### Unique molecular indexing (UMI)

UMI strategies have been shown to improve rare mutation calling in liquid biopsies. The increased duplication rates due to the high number of PCR amplification cycles to overcome cfDNA input limitations were reduced by the implementation of UMIs in lcWGS and the panel sequencing protocol (Additional file 1: Fig. S2F-G and Fig. S4E-F). The greater amount of input material used for the panel libraries (> 4 ng) than for the lcWGS (100 pg) libraries resulted in fewer duplicated fragments. The UMI deduplication workflow increased the average mean depth of coverage by 7% for lcWGS and the on-target mean depth of coverage of panel sequencing by threefold. The increase in mean depth of coverage was positively correlated with the MarkDuplication rate according to both lcWGS (Additional file 1: Fig. S2G) and panel sequencing (Additional file 1: Fig. S4F).

### *In silico* size selection, fragment length analysis and ctDNA estimation score

Paired-end sequencing reads allowed inference of the fragment lengths of cfDNA templates via the genomic locations of both ends after alignment to the reference genome. Samtools [34] was used to select paired reads with fragment lengths between 50 and 150 bases. Read retention after in silico size selection was quantified by comparing the number of properly paired primary alignments before and after filtering using samtools view. Secondary and supplementary alignments were excluded, whereas duplicate and QC-failed reads were retained to avoid bias in cfDNA fragment representation. These size-selected samples were used to call copy-number alterations via ichorCNA, where the PoN was created from this size-selection process. Tumor fractions before and after fragment size selection were compared cohort-wide using ichorCNA-derived tumor fraction estimates. To evaluate potential fragment length bias across CNV states, segment-level log2 ratios before and after size selection were compared across loss, neutral, gain, and amplification regions. Fragment length analysis and ctDNA quantification were performed using the cfdnakit R package.

An R package, cfdnakit, was developed to employ fragment length information from lcWGS data and was published as a Bioconductor package [35]. Briefly, cfDNA fragment counts were computed per 1 Mb non-overlapping genomic bins after excluding reads overlappingDUKE and DAC blacklisted regions [36] and (the) centromere(s). The short-to-long fragment ratio (SLRatio) — defined as the ratio of fragments of 100–150 nt to fragments of 151–250 nt — was calculated for each bin and corrected for guanine-cytosine (GC) content and mappability using locally estimated scatterplot smoothing (LOESS) regression, then normalized against a process-matched PoN. Bins were segmented using circular binary segmentation (CBS) [37], yielding a median z-score per segment (Z_segment_). Full derivation of the SLRatio formula and bin-level correction procedure are provided in Additional file 2. The ctDNA Estimation Score (CES) integrates genome-wide fragmentation patterns with copy number segmentation to quantify ctDNA burden in a single continuous metric. It is defined as:

$$\:CES=\:\frac{\sum\limits_{i=1}^{{N}_{segment}}\left(\left|{Z}_{{segment}_{i}}\times\:\:{l}_{{segment}_{i}}\right|\right)}{{N}_{segment}}\cdot\:SLRatio$$

where $\:{Z}_{{segment}_{i}}$ is the z score of segment,

$\:{l}_{{segment}_{i\:}}$ is the number of bins in$\:{segment}_{i}$.

and $\:{N}_{segment}$ is the total number of segments,

the *SLRatio* is the short-to-long ratio of the sample (See Supplementary Method).

By weighting segment-level fragmentation z-scores by their genomic length and scaling by the sample-wide SLRatio, CES captures both the magnitude and extent of tumor-associated fragmentation changes across the genome.

### CES cross-validation and classification performance

To confirm that CES performance was not driven by overfitting to the discovery cohort, we evaluated its ability to distinguish cancer patients from healthy controls using repeated stratified 10-fold cross-validation (100 repeats) and leave-one-out cross-validation (LOOCV), applied to 132 plasma lcWGS cancer samples and 10 healthy controls. In each cross-validation fold, an optimal CES threshold was derived from the training partition using Youden’s J statistic and applied to the held-out samples to estimate sensitivity and specificity. Mean receiver operating characteristic (ROC) curves and 95% confidence intervals were derived across all folds and repeats. LOOCV served as an independent confirmation, with each sample classified using a threshold learned from all remaining samples. All analyses were performed in Python (v3.12) using scikit-learn (v1.5) [38] and SciPy (v1.14) [39].

### CES detection threshold and benchmarking against orthogonal ctDNA metrics

A CES positivity threshold of 3 was defined as the upper boundary of the healthy control distribution, prioritizing specificity. An alternative threshold based on Youden’s J statistic is also reported for settings where sensitivity is preferred. To benchmark CES against independent evidence of ctDNA, samples were considered orthogonal-positive if ctDNA was detectable by at least one non-CES method (ichorCNA tumor fraction ≥ 3%, or variant allele frequency (VAF) > 0 by WES or panel sequencing). ROC curves were generated for CES, SLRatio, and ichorCNA tumor fraction against this orthogonal ground truth. Spearman correlations between CES and each orthogonal metric were computed in samples with detectable signal to avoid zero-inflation bias.

### Whole-exome sequencing analysis

Similar to WGS analysis, the OTP service was used to align WES sequencing data and call somatic variants. Picard CollectWgsMetrics, CollectOxoGMetrics, and CollectInsertSizeMetrics estimate the average depth of sequencing coverage, the error rate from the oxidation of guanine to 8-oxoguanine, and the distribution of template sizes according to on-target reads. The samples were subjected to quality thresholds of mean depth of coverage at 60x, and an average Phred score of 30 was calculated from the substitutions C(C > A or G > T). Somatic SNV and indel calling from matching tumor/control WES were processed via the ODCF in-house SNVCallingWorkflow and IndelCallingWorkflow through the OTP service [40]. The minimum confidence score was set to 8. Nonsynonymous somatic mutations were selected for downstream analysis. The same workflows were also used for somatic SNV and indel calling from matching cfDNA and healthy controls with the following options: -t 500 -c 0 -x 1 -l 1 -e 0 to allow detection with a low allelic fraction. We used the tumor VCF file containing high-confidence nonsynonymous somatic variants as a reference to interrogate variant abundance in cfDNA from patients. An in-house bash script was used to annotate the existence of a tumor variant allele by looking up read pileup information from cfDNA. We considered read pileup information with a minimum read mapping quality of 1 and a base quality of 20. Tumor variants were reported to be present in the cfDNA sample if at least one read supported the tumor allele and if at least 11 reads covered the affected genomic region. We used PureCN [41] for CNV calling on WES data. To create a PoN, we selected cfDNA WES samples with on-target mean depth of coverage between 142 and 269. Subsequently, cfDNA samples from patients with fewer than three tumor variants were included. BAM files of selected samples were processed by GATK Mutect2 [42] in tumor-only mode for calling both germline and somatic variants with the setting --max-mnp-distance 0 --min-base-quality-score 20 --annotation BaseQuality --read-filter MappingQualityReadFilter --read-filter OverclippedReadFilter --minimum-mapping-quality 30 --read-filter FragmentLengthReadFilter --min-fragment-length 30. After that, we combined VCF files with GATK CombineVariants [42] with the option --minimumN 3. This setting retained only variants commonly found in at least three samples. Finally, PureCN CNV calling was applied to cfDNA WES samples with the following settings to (also) allow the detection of CNV in samples with lower tumor purity: --minpurity 0.05 --minaf 0.01 --error 0.0005 --maxploidy 3 --maxcopynumber 8 --padding 25 --model betabin --funsegmentation PSCBS –postoptimize.

### Panel sequencing analysis

Sequence alignment was performed via the ODCF in-house bioinformatics workflow. The workflow aligns sequencing reads to the human reference genome GRCh37 (hg19) plus the PhiX contig. As described in the WGS section, fgbio (v1.1.0; Fulcrum Genomics) and Picard were used to manipulate BAM files and call molecular consensus reads from UMI information. Picard SamToFastq extracted consensus reads into FASTQ format. These consensus reads were aligned to the reference genome again by using BWA-MEM without marking duplicate reads. Picard CollectWgsMetrics and CollectOxoGMetrics (v2.21.8) were used to estimate the sequencing quality, including the average depth of sequencing coverage and level of pre-PCR artifacts from the oxidation of guanine to 8-oxoguanine of on-target reads. For WES, tumor nonsynonymous somatic variants were used as a reference to look for concordance with the cfDNA of patients. Read pileup information from reads with a minimum mapping quality of 1 and a minimum base quality of 20 was included. Tumor variants were considered to be represented in cfDNA if at least one read supported the tumor allele at a minimal mean depth of coverage of 11.

### Structural variant analysis

Genomic structural variants (SVs) were detected using SOPHIA [43] as implemented in the DKFZ structural variation calling workflow. SOPHIA identifies candidate SVs based on supplementary alignments generated by BWA-MEM, which indicate discordant read mappings consistent with underlying genomic rearrangements. SV calling was performed for each sample against the matched control. Resulting SV calls were filtered using the SOPHIA output annotations. For downstream analysis, only events with an eventScore ≥ 3 were retained. Additional filtering removed likely artefacts, including events involving decoy contigs (hs37d5), ambiguous SV annotations, and sex chromosome cross-mapping events (X–Y and Y–X). For visualization and comparison across samples, filtered SV call sets were combined and plotted using the R package *circlize *[44]. Breakpoint coordinates were extracted and converted to UCSC-style chromosome notation, and SVs were visualized as genomic links on a circular ideogram. Each sample was displayed in a distinct semi-transparent color to allow comparison of shared and private rearrangements. Samples without SVs passing the filtering criteria were reported separately as empty outputs.

### Data visualization and statistical analysis

Patient data management was performed via GraphPad Prism 8 (GraphPad, Software, Inc., La Jolla, CA, USA). Cohort representation diagrams and tumor-informed oncoplots were visualized via ComplexHeatmap [45]. Nonparametric comparisons between groups were performed with the Wilcoxon rank-sum test and the Kolmogorov–Smirnov test in the R environment (v4.0.0). Correlation analyses were performed using Spearman rank correlation unless otherwise stated. P values of < 0.05 were considered statistically significant, with p values represented as follows: * *p* < 0.05, ** *p* < 0.01, *** *p* < 0.001, and **** *p* < 0.0001. “ns” denotes differences in means that were not significant. All error bars shown represent the standard deviation unless otherwise stated. Graphical illustrations of patient disease courses were created with BioRender.com.

## Results

### Characteristics of the INFORM liquid biopsy cohort

**Fig. 1:**
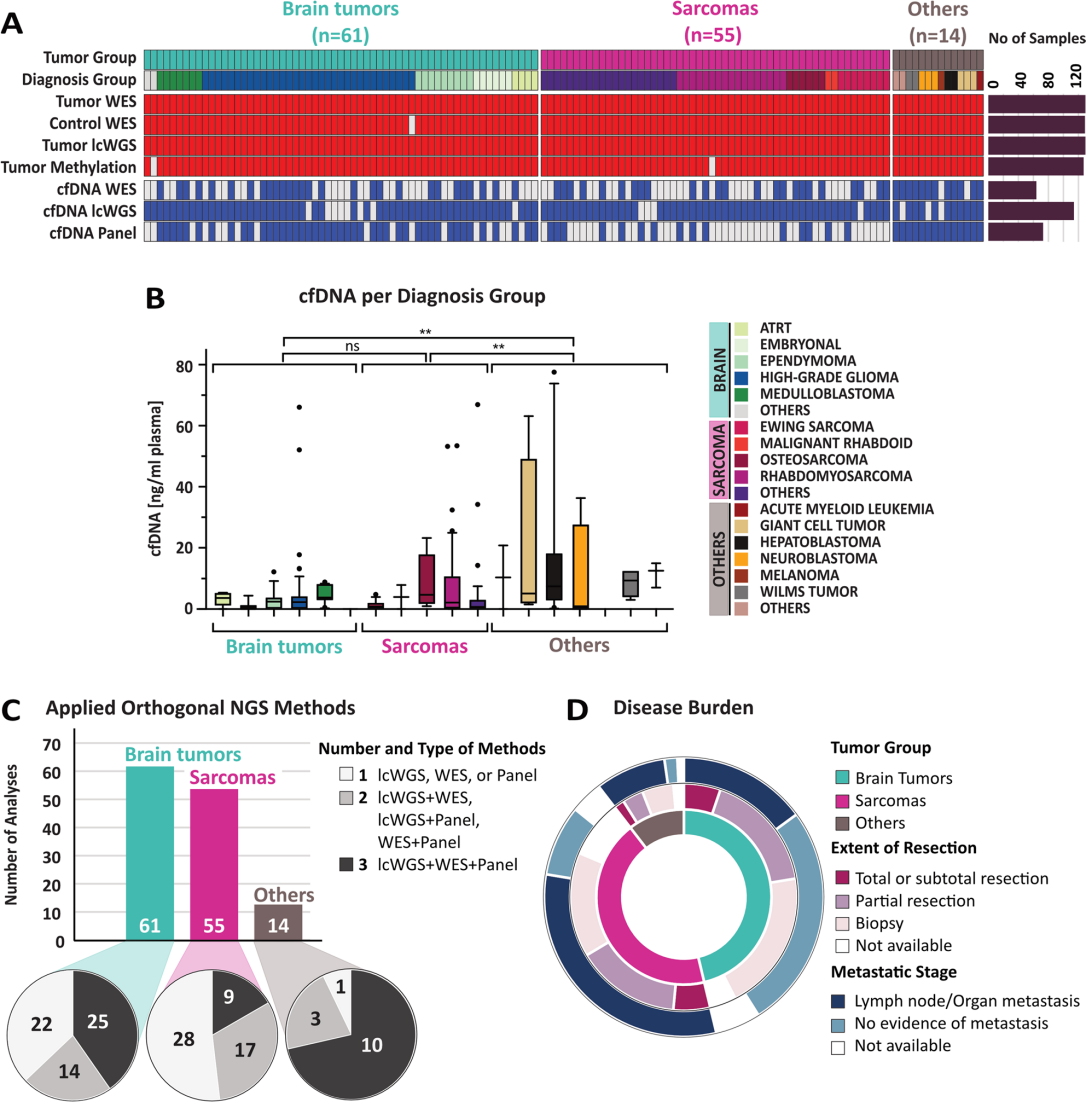
INFORM Liquid Biopsy Cohort Characteristics. **A** Oncoprint indicating DNA methylation-based diagnosis groups and molecular analyses performed on tumor, blood (control) and plasma-derived cell-free DNA (cfDNA) for INFORM patients with available liquid biopsy material (*n* = 130). **B** Amounts of cfDNA (ng/ml plasma) isolated from plasma of the indicated diagnosis group. Kruskal-Wallis test (*p* = 0.0027) followed by Dunn’s multiple comparisons test (*p* > 0.9999 for brain tumors vs. sarcomas; *p* = 0.0025 for brain tumors vs. others; *p* = 0.0067 for sarcoma vs. others). **C** Available orthogonal next generation sequencing (NGS) analyses (low-coverage whole-genome sequencing (lcWGS), whole-exome sequencing (WES) and panel sequencing (panel)) of cfDNA are depicted allowing for two- (*n* = 34) or three-way (*n* = 44) comparisons within respective tumor groups. **D** Overview of disease burden: tumor group (inner ring), extent of resection (middle ring) and metastatic stage (outer ring)

The study cohort included 130 pediatric patients enrolled in the INFORM registry between 2015 and 2019, each providing at least one plasma-based liquid biopsy during the current disease episode (Median 5 days to surgery, Fig. 1A, Additional file 1: Fig. S1A–B). Molecular profiles of tumor tissues were generated using the INFORM pipeline, based on matched tumor samples and germline controls derived from peripheral blood cells. This included 130 tumor-normal WES pairs, 130 lcWGS tumor-normal pairs, and 128 tumor methylation array profiles (Fig. 1A). The cohort’s demographic, and sampling characteristics, including time to surgery, the number of samples collected per year, and age at enrollment stratified by tumor group, are summarized in Additional file 1: Fig. S1B–D. Of the cohort, 61 patients had brain tumors, 55 had sarcomas, and 14 had other solid malignancies (Fig. 1A). Among 138 initial patients, 8 were excluded due to inclusion criteria failure or tissue quality issues, leaving 130 patients (94%) for analysis via at least one sequencing approach, regardless of plasma volume or cfDNA yield (Additional file 1: Fig. S1A). cfDNA was extracted from all plasma samples, and its quantity was assessed based on the signal of the first three nucleosomal peaks (160–500 bp) derived from Bioanalyzer profiles, which served as a surrogate for cfDNA abundance (Fig. 1B). In addition, the total amount of isolated DNA (Additional file 1: Fig. S1E), and cfDNA purity, expressed as the proportion of cfDNA relative to total DNA, were assessed (Additional file 1: Fig. S1F). Higher cfDNA levels were observed in patients with sarcomas (IQR 22.4–68.0 ng/ml) and other solid tumors (IQR 49.2–79.6.0 ng/ml) compared to brain tumor patients (IQR 0.0–70.5 ng/ml; Fig. 1B). For the liquid biopsy cohort, cfDNA samples were analyzed to detect genetic alterations including CNVs, SNVs, and Indels, using lcWGS, WES, and targeted panel sequencing. Most cfDNA samples (35.7%, *n* = 55) could be analyzed using one method, while 33.8% (*n* = 44) and 26.2% (*n* = 34) were analyzable using three and two methods, respectively (Fig. 1C). The final cfDNA dataset included 132 lcWGS analyses (116 patients), 66 WES analyses (64 patients), and 74 panel sequencing analyses (73 patients) (Fig. 1A). Stringent quality criteria defined for all three sequencing platforms were applied, filtering out 4% of cfDNA analyses (4 lcWGS, 5 WES, 3 panel; Additional file 1: Fig. S1G). CNV profiles were compared across collection centers and no major differences between collection centers were observed, suggesting consistent data quality across sites (Additional file 1: Fig. S1H). Disease burden potentially affecting liquid biopsy detection is shown in Fig. 1D.

### The CtDNA estimation score (CES) enables sensitive tumor signal detection from short-fragment data

To overcome volume limitations in pediatric liquid biopsy cohorts, we optimized the workflow, including improved preanalytical protocols for higher cfDNA yields and purity [19]. Adjustments to the WGS library protocol allowed for molecular profiling of plasma samples with cfDNA eluate concentrations below the detection limit (50 pg/µl) (Additional file 1: Fig. S2A–C). Optimized library preparation and tailored amplification cycles allowed detection of plasma-based CNV profiles, even with low cfDNA input amounts (median 100 pg, mean 136 pg; Additional file 3: Table S1 and Additional file 1: Fig. S2D–E). UMI strategies reduced amplification bias, increasing the mean depth of coverage by 8.6%, from 1.27 to 1.38, for lcWGS (Fig. S2F). The fold change in mean depth of coverage was positively correlated with the duplication rate (Additional file 1: Fig. S2G).

**Fig. 2:**
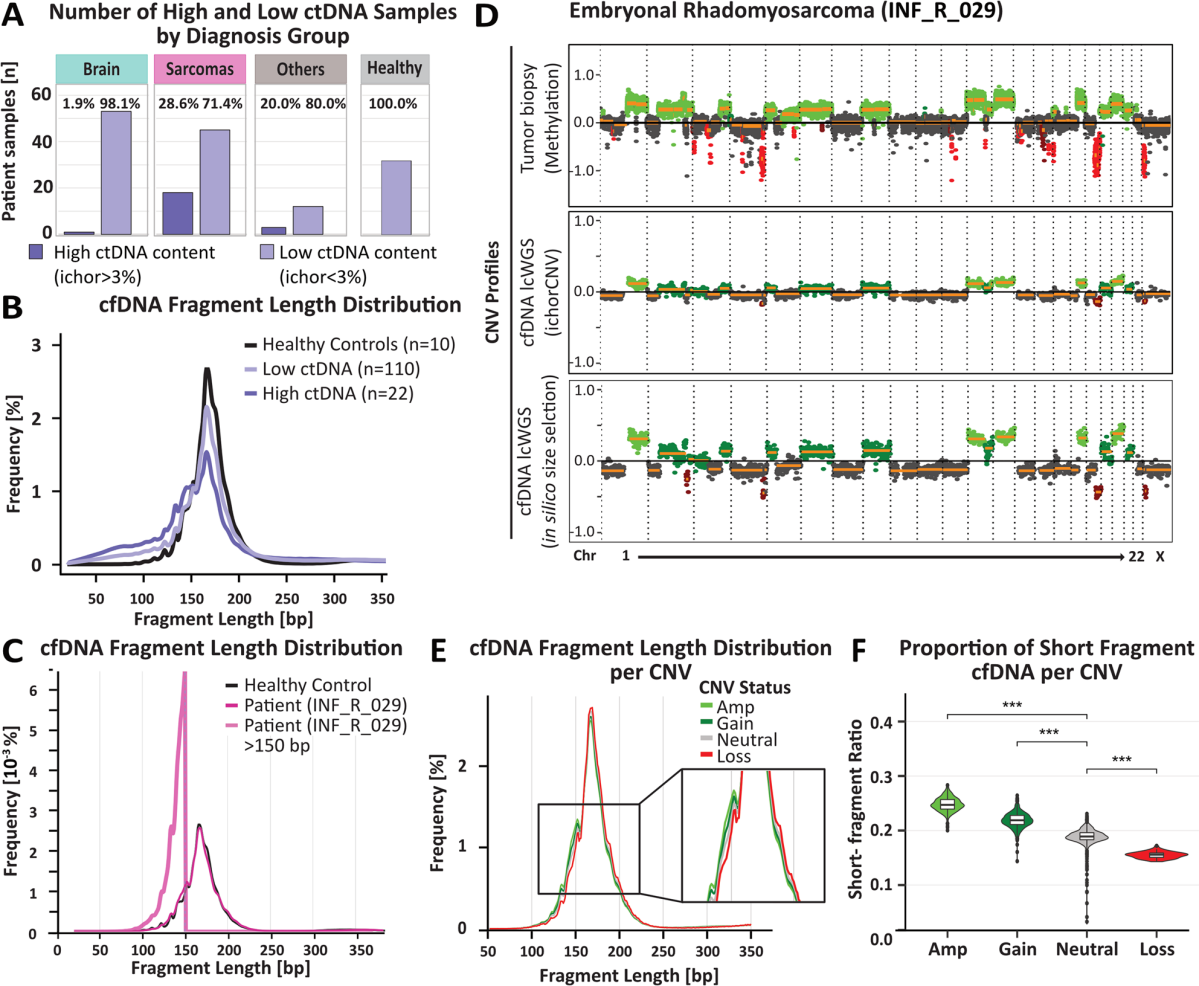
Bioinformatic Refinement of Liquid biopsy-based Low-coverage Whole-Genome Sequencing (lcWGS) to Enhance Tumor Detection from Plasma **A** Absolute and relative distribution of brain tumor, sarcoma, and other tumor cell-free DNA (cfDNA) samples with high (ichor > 3%, dark purple) or low (ichor < 3%, light purple) ctDNA fractions based on ichorCNA. **B ***In silico* fragment-length distribution of plasma-derived cfDNA from patients with high (ichor > 3%, dark purple) or low (ichor < 3%, light purple) ctDNA fractions plotted against healthy controls (black). **C** In silico-determined fragment length distribution of cfDNA from a patient tumor (pink, corresponding copy number variation (CNV) plots in (D) compared to pool of cfDNA of healthy controls (*n* = 10, black). **D** Genome-wide CNV profiles of a rhabdomyosarcoma tumor derived from DNA methylation array analysis (upper panel), of a plasma-derived cfDNA sample from the same patient analyzed by lcWGS with the ichorCNA standard pipeline (mid panel) or with in silico short fragment (100–150 bp) filtering for improved tumor detection (lower panel). **E** In silico-determined fragment length distribution in relation to the indicated copy-number status. **F** Violin plot of short-fragment ratio in relation to copy-number aberrations for plasma-derived cfDNA of tumor patient INF_R_029 from C. Compared to CNV neutral regions (grey), the short-fragment ratio was significantly elevated in genomic regions with gains (3 N, green) or focal amplification (> 3 N, light green) and significantly decreased in genomic regions with losses (red). *P* values were calculated by Wilcoxon rank sum test with *** = *p* < 0.001

Using the reported 3% detection limit for the ichorCNA pipeline [28], we classified patients into high (ichor ctDNA fraction ≥ 3%) or low ctDNA (ichor ctDNA fraction < 3%) (Additional file 3: Table S1). Tumor detection rates with ichorCNA were 1.9% for brain tumors, 28.6% for sarcomas, and 20.0% for other malignancies, with 83.3% of samples below the detection limit (Fig. 2A and Additional file 1: Fig. S2H). Recognizing that low cfDNA input is a common challenge in pediatric oncology, previous studies have demonstrated that tumor-derived signals can still be detected at sub-nanogram input levels, and that the 3% ichorCNA threshold remains clinically informative [20, 46, 47]. Nevertheless, we applied this threshold as a conservative reference, and results near this cut-off were interpreted with caution. Given these limitations of ichorCNA-based detection, we incorporated cfDNA fragment length as an additional ctDNA indicator. Concordant with previous reports [48], shorter fragments were found most abundant in high-ctDNA samples (mean length 164.5 bp) compared to low-ctDNA samples (177.4 bp) and healthy controls (179.6 bp, Fig. 2B). By applying in silico size selection for cfDNA fragments smaller than 150 bp (Fig. 2C), we observed a marked increase in CNV amplitudes (Fig. 2D). Cohort-wide analyses further demonstrated that, although size selection reduced total read counts (Additional file 1: Fig. S2I), it led to a net increase in ctDNA signal in most samples (Additional file 1: Fig. S2J). Specifically, only a minority of cases showed a decrease in tumor fraction after size selection, indicating that enrichment of tumor-derived fragments generally outweighs the loss of reads (Additional file 1: Figs. S2J–K). The short-to-long-fragment ratio strongly correlated with copy number states, with short fragments enriched in gained and amplified regions (Figs. 2E–F and Additional file 1: Fig. S3A–B). Building on this observation, we developed the CES, which integrates in silico size selection of cfDNA fragments with copy number–informed weighting to improve tumor signal detection. This combined approach enhanced separation of copy number states and increased the overall detectability of tumor-derived cfDNA. (Method section, Supplementary Methods). Additional benchmarking analyses further supported the biological and technical robustness of CES (Additional file 1: Fig. S3). Importantly, CES values were comparable between adult and pediatric healthy controls (Additional file 1: Fig. S3C) and showed clear separation from tumor samples (Additional file 1: Fig. S3D). Across the different control cohorts, CES demonstrated consistently high classification performance, with combined area under the curve (AUC) values above 0.94 and stable sensitivity and specificity, indicating robustness to cohort composition (Additional file 1: Fig. S3E). Cross-validation analyses demonstrated high and stable performance, with an AUC of 0.96 (Additional file 1: Fig. S3F), indicating that the model is not driven by overfitting. Samples that were not correctly classified showed CES values within the healthy control range and low ctDNA fractions (Additional file 1: Fig. S3D), consistent with tumor levels near the detection limit. To define a clinically interpretable threshold, we evaluated CES distributions in healthy controls (adult) and established a cut-off of 3.29 based on cross-validation (Additional file 1: Fig. S3G–H). At this threshold, CES achieved high sensitivity across tumor types, with lower sensitivity observed in brain tumors, consistent with their generally lower ctDNA shedding (Additional file 1: Fig. S3G–H). Together, these results demonstrate that integrating fragment size information with copy number analysis through CES improves plasma-based tumor detection and provides a robust and generalizable framework for cfDNA analysis.

### CES increases sensitivity and specificity of tumor detection in the inform liquid biopsy cohort

**Fig. 3:**
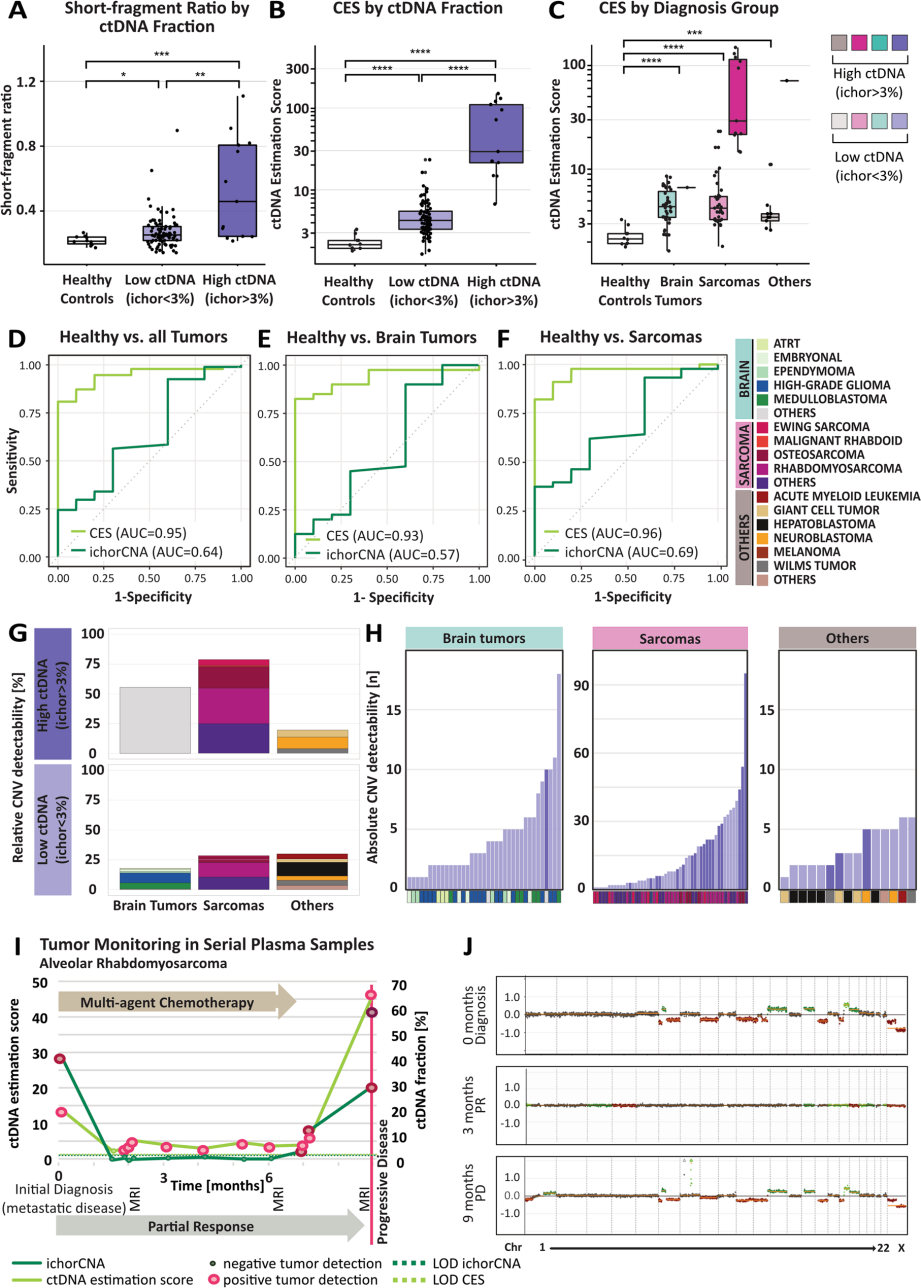
Clinical Validation of Low-Coverage Whole-Genome Sequencing (LcWGS) in a Pediatric Pan-Cancer Liquid Biopsy Cohort. **A** Short-to-long fragment ratio in liquid biopsy plasma samples of healthy controls, pediatric cancer patients with low (ichor < 3%) or high cell-free tumor DNA (ctDNA) fraction (ichor > 3%) based on ichorCNA [28]. *P*-values were calculated by Wilcoxon test with *= *p* < 0.05** = *p* < 0.01, *** = *p* < 0.001. Kruskall-Wallis, *p* = 9.4 × 10^− 4^. **B** CtDNA estimation score (CES) distribution across the same pediatric pan-cancer cohort as in A) grouped by ctDNA fraction and compared to healthy controls. *P*-values were calculated by unpaired t-test with **** = *P* ≤ 0.0001. Kruskall-Wallis, *p* = 6.2 × 10^-11^. **C** CES distribution across the same pediatric pan-cancer cohort as in (A) and (B) grouped by both diagnosis group and ichorCNA ctDNA fraction. **D-F** Receiver Operating Characteristic (ROC) curves illustrating increased sensitivity and specificity of CES to ichorCNA ctDNA fraction analyses for **D** healthy controls versus pan-cancer cohort (Area under the curve (AUC) CES = 0.95, ichorCNA = 0.64;); **E** versus brain tumor patients (AUC CES = 0.93, ichorCNA = 0.57), and **F** versus sarcoma patients (AUC CES = 0.96, ichorCNA = 0.69). **G-H** Bar plots showing **G** relative and **H** absolute numbers of detected tumor tissue CNVs in plasma cfDNA per diagnosis group. Plasma samples with ctDNA fraction ichor ctDNA fraction < 3% are labeled in light purple, plasma samples with ctDNA fraction ichor ctDNA fraction > 3% are labeled in dark purple. **I** IchorCNA ctDNA fraction and CES of cfDNA from an alveolar rhabdomyosarcoma patient collected over 9 months. Red circles indicate positive tumor detection, highlighting the detection of minimal residual disease (MRD) 2 months prior to magnetic resonance imaging (MRI) diagnosis of progressive disease. **J** Genome-wide CNV profiles of plasma cfDNA from rhabdomyosarcoma patient collected serially during therapy course (PR-partial response, PD-progressive disease)

We categorized the liquid biopsy samples into two groups based on ctDNA content: high ctDNA fraction (ichor > 3%) and low ctDNA fraction (ichor < 3%). We then calculated the ratio of short fragments (100–150 bp) to long fragments (151–250 bp) [48]. This ratio was significantly higher in cancer patients compared to healthy controls. Patients with a high ctDNA fraction had a median short-to-long fragment ratio of 0.43 (*p* = 0.03), and those with a low ctDNA fraction had a median short-to-long fragment ratio of 0.22 (*p* = 0.00064), while healthy controls had a median short-to-long fragment ratio of 0.18 (Fig. 3A). The highest short-to-long fragment ratios were found in sarcoma patients (Additional file 1: Fig. S4A–B). Notably, in the low ctDNA group, only in silico size selection resulted in a significant improvement in tumor detection, whereas in the high ctDNA group, both UMI-based error correction and in silico size selection significantly increased the estimated ctDNA fraction (Additional file 1: Fig. S4C–D). The magnitude of improvement across diagnostic subgroups for both low and high ctDNA settings is detailed in Additional file 1: Fig. S4E–F. CES significantly improved tumor detection, particularly in brain tumor patients, reaching levels similar to those of solid tumors (Fig. 3B-C). The CES demonstrated higher accuracy for tumor detection than ichorCNA, with AUC values of 0.95 versus 0.64 overall (Fig. 3D) and 0.93 versus 0.57 for brain tumors (Fig. 3E) and 0.96 versus 0.69 for sarcomas (Fig. 3F).

Expanding the analysis beyond tumor detection, we assessed the concordance between plasma cfDNA and matched tumor CNV profiles using lcWGS data, focusing on clinically relevant CNVs from the INFORM actionable targets list. CNV detectability was defined as the proportion of tumor-derived CNVs that could be identified in the matched plasma cfDNA sample (i.e., number of concordant CNVs detected in cfDNA divided by the total number of CNVs identified in the tumor tissue). Among brain tumors, the only high-ctDNA sample came from a medulloblastoma patient with metastatic spread (M4) and residual tumor mass (R4), showing strong agreement between cfDNA and tumor-derived CNV profiles, with 55% (10/18) of tumor CNVs detected (Fig. 3G (upper left) and 3H (dark purple)). In low-ctDNA brain tumors, high-grade gliomas and medulloblastomas had the highest CNV detectability (Fig. 3G (lower left) and 3H (light purple)). Sarcomas in the high-ctDNA group had the highest CNV detection rate (79%; Fig- 3G, upper middle), with osteosarcoma, rhabdomyosarcoma, and other sarcomas showing similar detection rates across high (74%) and low (28%) ctDNA groups (Fig. 3G, middle, Fig. 3H). Among other tumors, neuroblastomas dominated successful CNV detection in high-ctDNA cases (Fig. 3G, upper right), while low-ctDNA tumors had comparable CNV detection rates (30%) to sarcomas (Fig. 3G, lower right). Notably, sarcoma plasma CNV profiles in high-ctDNA cases closely mirrored tumor tissue alterations, reinforcing their clinical relevance (Additional file 1: Fig. S4G).

Finally, we assessed longitudinal tumor monitoring using serial plasma samples (*n* = 12) from a patient with metastatic alveolar rhabdomyosarcoma (Fig. 3I-J). Following an initial partial response (PR) to multi-agent chemotherapy, residual tumor was monitored through routine radiologic imaging. While ctDNA fractions remained below detection limits (Fig. 3I, ctDNA fraction—dark green), the CES score effectively captured the remaining tumor burden (CES—light green). Seven months post-diagnosis, CES signaled progressive disease (PD), detecting it two months earlier than routine imaging performed at three-month intervals (Fig. 3I-J). To further validate the clinical relevance of the CES score, we analyzed a previously published longitudinal cfDNA dataset from a patient with Ewing sarcoma [49]. A total of six cfDNA samples were collected across the clinical timeline, including diagnosis, treatment, and relapse phases. As depicted in the time resolved analyses, tumor-derived signal in cfDNA increased substantially during relapse periods with ichorCNA-estimated ctDNA fraction rising from 0.09 at diagnosis to 0.55 at first relapse, and remaining elevated at 0.42 during the second relapse (Additional file 1: Fig. S5A). In parallel, the CES score showed a concordant pattern with disease progression remaining low at diagnosis, peaking at first relapse, transiently declined following treatment, and rose again at secondary relapse (Additional file 1: Fig. S5B and Fig. S5C). Notably, it remained elevated prior to the patient’s death on Day 654.

These findings underscore that the CES score mirrors clinical tumor burden and aligns with established measures such as ichorCNA ctDNA fraction. This demonstrates the potential of CES as sensitive, non-invasive biomarker for real-time monitoring of therapy response in the future in pediatric cancers with low mutational burden.

### Whole-exome and targeted panel sequencing for detecting druggable mutations in cfDNA

**Fig. 4:**
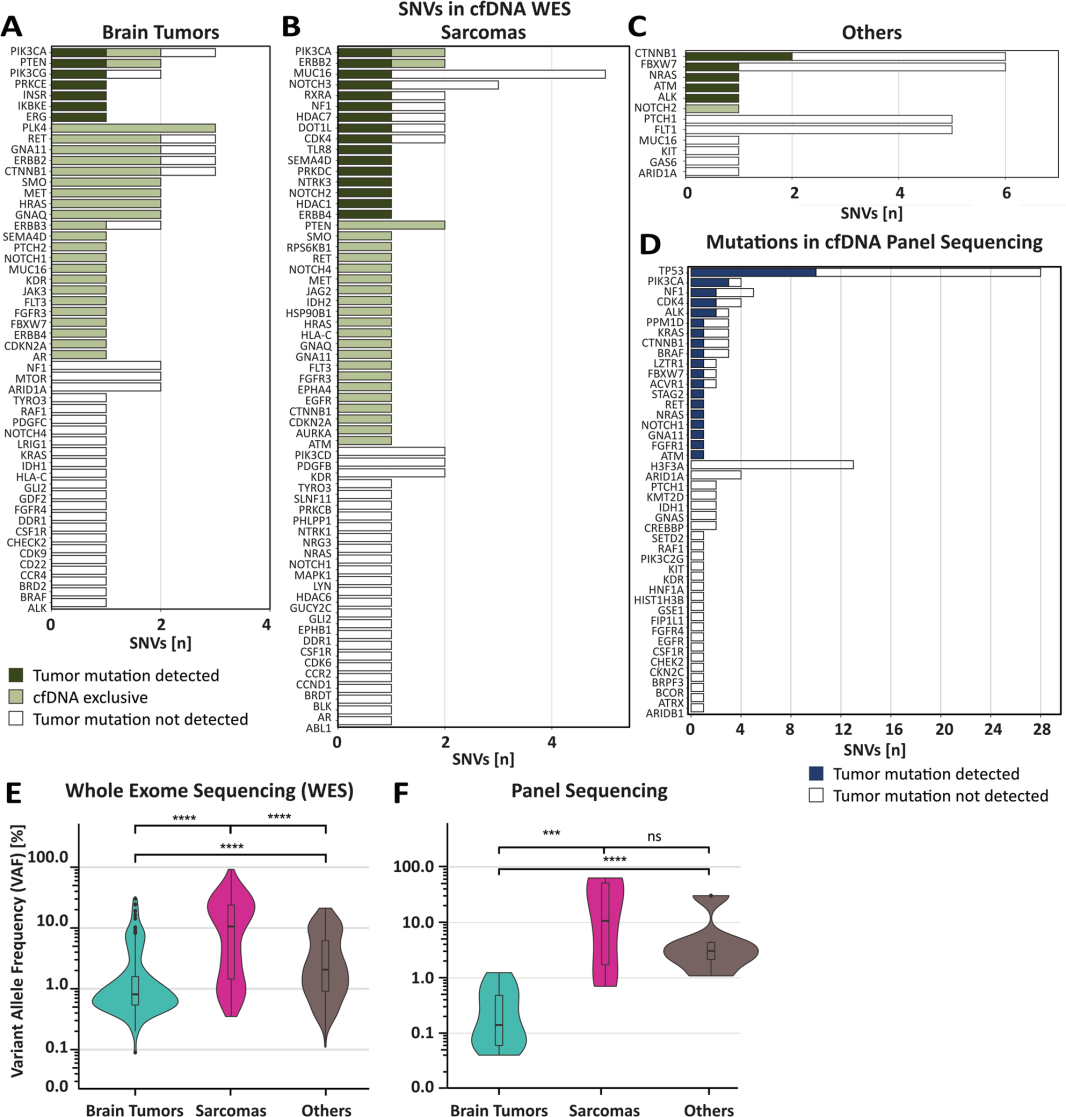
Whole-Exome Sequencing (WES) and Targeted Panel Sequencing Approaches Complemented Low-Coverage Whole-Genome Sequencing (LcWGS) through Detection of Single Nucleotide Variants (SNVs). **A** SNVs detected in cell-free DNA (cfDNA) of **A** brain tumor, **B** sarcoma, and **C** other tumor patients by WES. **D** SNVs detected by targeted panel sequencing. Color coding indicates detection status: WES, dark green - somatic tumor SNVs detected in both tumor and cfDNA, light green - cfDNA-exclusive SNVs, white- tumor-exclusive SNVs. Panel sequencing, blue - SNVs detected in both tumor and cfDNA, white - tumor-exclusive SNVs. **E-F** Variant allele frequency (VAF) of SNVs detected in cfDNA for indicated diagnostic groups **E** by WES and **F** by panel sequencing

Beyond CNV-based tumor detection, liquid biopsies in personalized oncology require mutation analysis to assess target abundance and detect resistance early. We applied WES and targeted panel sequencing to cfDNA samples with sufficient material, enabling higher mean depth of coverage and analysis (Additional file 1: Fig. S6). WES-based detection of somatic SNVs was assessed in 28 brain tumors, 26 sarcomas, and 12 other pediatric cancers, focusing on 367 clinically relevant genes (Additional file 4: Table S2, Fig. 4 and Additional file 1: Fig. S6A-D). Average detection rates varied across tumor types (brain tumors: 11.3%, sarcomas: 32%, others: 27%; Fig. 4A-C). Patient-specific alterations are shown for each diagnosis group as both absolute counts and relative proportions of tumor-informed cfDNA SNVs, emphasizing the detection rates of clinically relevant mutations (Additional file 1: Fig. S6B–D). Among sarcomas, 8 cfDNA samples had druggable alterations, 67% matching tumor tissue results, while 33% contained additional cfDNA-exclusive SNVs (Fig. 4B and Additional file 1: Fig. S6C).

For panel sequencing, UMI barcoding increased read recovery threefold (median depth of coverage: 328.5 to 820.5) (Additional file 1: Fig. S6E), enhancing SNV detection at lower VAF (Additional file 1: Fig. S6F–G). The most recurrent SNVs in this cohort were in *TP53*, *PIK3CA*, *NF1*, *CDK4*, and *ALK* (Fig. 4D). Reducing the panel to 130 genes [22] improved relative detection rates across all groups (25–100%, Additional file 1: Fig. S6H–J), with tumor-informed SNVs found in 21% of brain tumors (Additional file 1: Fig. S6H), 71% of sarcomas (Additional file 1: Fig. S6I), and 40% of other tumors (Additional file 1: Fig. S6J). Overlap of cfDNA-detected SNVs across tumor groups is shown in a Venn diagram (Additional file 1: Fig. S6K), highlighting both tumor-type–specific and shared mutational profiles. Panel sequencing demonstrated increased sensitivity for low-VAF SNVs in brain tumors, detecting variants down to a minimum VAF of 0.04% compared to 0.09% with WES (Fig. 4E–F). On-target coverage was comparable across the major diagnosis groups analyzed by panel sequencing (Additional file 1: Fig. S6L), suggesting that differences in sequencing depth are unlikely to account for this observation. Instead, the increased sensitivity is likely attributable to the panel design, which is enriched for brain tumor–relevant genes. Consequently, brain tumors, where variants are often present at low allele frequencies, benefited most from the increased sensitivity. In contrast, no additional sensitivity gain was observed in sarcomas (median VAF (mVAF): WES 14.69% vs. panel 24.58%) or other tumor entities (mVAF: WES 4.19% vs. panel 7.34%), where variants were generally detected at higher VAFs. Together, these findings indicate that panel sequencing is particularly advantageous for detecting low-frequency variants in tumor types for which the panel is specifically designed.

### Performance comparison of cfDNA sequencing approaches

**Fig. 5:**
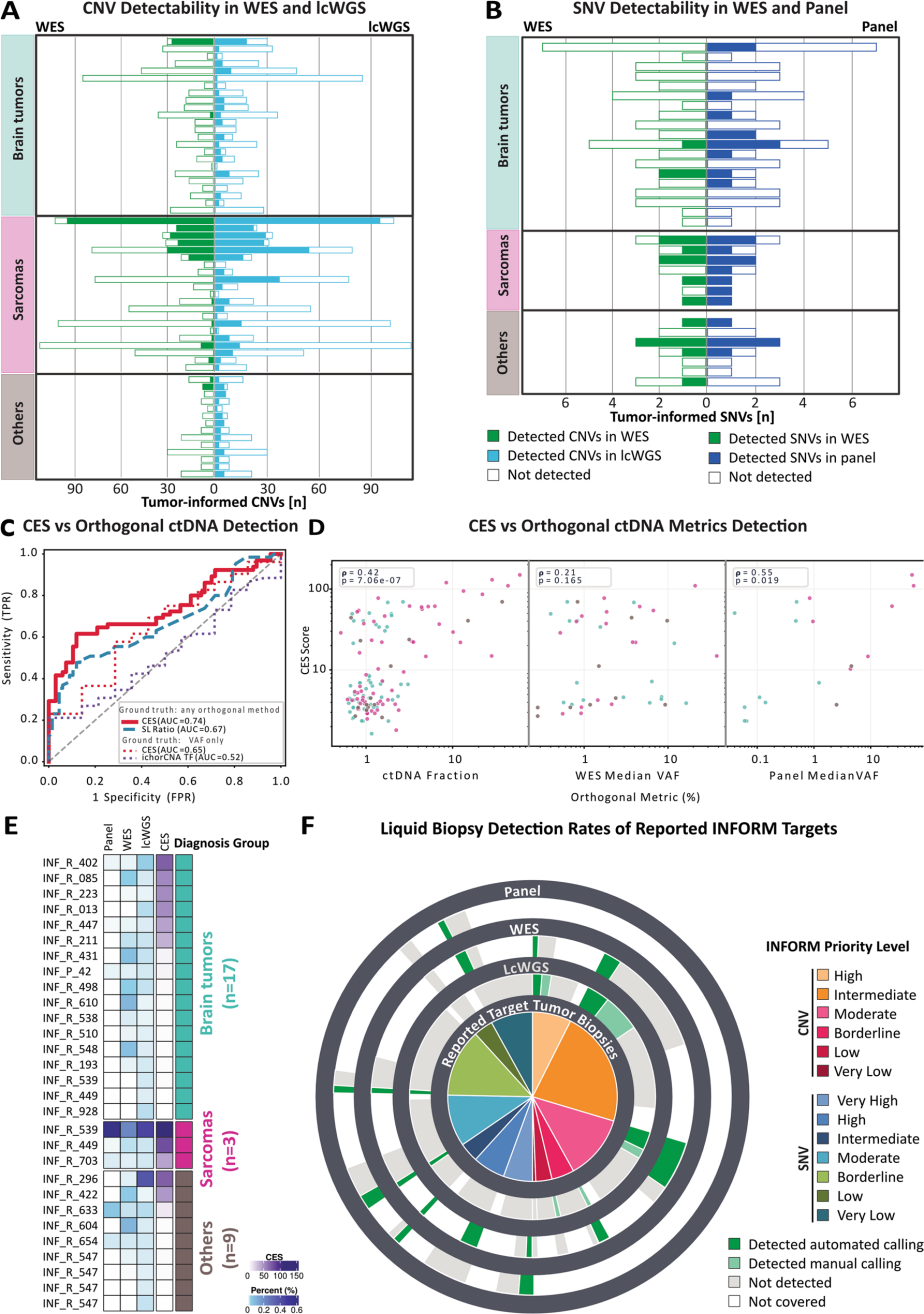
Comparison of Orthogonal NGS Approaches on cell-free DNA (cfDNA) Liquid Biopsies. **A** Orthogonal comparison of copy number variation (CNV) detectability in liquid biopsy whole-exome sequencing (WES) (left bars) versus low-coverage whole-genome sequencing (lcWGS) (right bars) in matched patient plasma samples. Green – tumor CNVs detected in cfDNA WES, light blue – tumor CNVs detected in cfDNA lcWGS, white – tumor CNVs not detected by the respective method. **B** Orthogonal comparison of single nucleotide variant (SNV) detectability in liquid biopsies in WES (left bars) versus panel sequencing data (right bars) in matched patient plasma samples. Green – common SNVs in tumor and cfDNA detected in cfDNA WES, dark blue – common SNVs in tumor and cfDNA detected in cfDNA lcWGS, white – tumor SNVs not detected by the respective method. **C** Receiver operating characteristic (ROC) curves comparing the performance of CES, the SLRatio, and ichorCNA tumor fraction in discriminating samples with independently confirmed ctDNA (orthogonal-positive) from samples without ctDNA detected by any orthogonal method. Dotted curves indicate performance against a VAF-only ground truth, used to avoid circularity arising from ichorCNA-based ctDNA detection. **D** Correlation of CES scores with orthogonal ctDNA detection metrics, including ichorCNA-derived tumor fraction (TF), WES-derived SNV VAF, and panel sequencing-derived SNV VAF. CES showed moderate correlation with panel VAF (ρ = 0.55) and ichorCNA TF (ρ = 0.42), but weaker correlation with WES VAF (ρ = 0.21), indicating that CES captures complementary cfDNA features beyond conventional ctDNA quantification metrics. **E** Direct comparison of liquid biopsy-based tumor detection ability by plotting CES, ichorCNA ctDNA fraction (%) in lcWGS, and median VAF (%) of tumor-informed mutations in WES or panel sequencing in matched samples of 29 patients. Diagnosis groups are indicated. Discrepancy between the three methods was observed for brain tumor patient 11, 15 and 16 (INF_R_431, INF_R_610, INF_R_548) and other tumor patients (INF_R_296, INF_R_604). **F** Sunburst plot summarizing cfDNA-based detection of targets (CNVs/SNVs) that were reported for corresponding tumors in the INFORM molecular tumor board. Target priority levels are indicated in the inner circle. Dark green – alteration called with automated bioinformatics pipeline, light green – alterations called through visual inspection, red – alteration not detected despite being covered in data of the indicated method, white – alteration not covered in data of the indicated method

We compared lcWGS, WES, and panel sequencing for detecting tumor-derived alterations in plasma-based liquid biopsies, using tumor tissue alterations detected by lcWGS (CNVs) or WES (SNVs) as ground truth (Fig. 5). For CNV detection, lcWGS showed a higher detection rate (≥ 1 CNV detected, 88% light blue) compared to WES (25%, dark green, Fig. 5A), with sarcomas exhibiting the highest overall detection rates (lcWGS 90%, WES 52%), followed by other pediatric cancers (lcWGS 100%, WES 14%) and brain tumors (lcWGS 79%, WES 8%). The number of tumor CNVs (median 18, maximum 291) did not affect detection rates. In terms of SNVs, WES identified more tumor-specific alterations than lcWGS (WES: 84%, lcWGS: 21%), with Indels detected in 42% of tumors by WES and 6% by lcWGS (Additional file 1: Fig. S7A and Fig. S7B). Given the low sequencing depth (mean ~ 3×) and limited cfDNA input (mean 139 pg), lcWGS is not intended for SNV detection and therefore does not provide a comparable basis to WES (130×, mean 41 ng input). Accordingly, WES identified the majority of somatic SNVs in liquid biopsies (965/4081), while lcWGS detected only a small subset (52/4081; Additional file 1: Fig. S7A), likely reflecting exceptional cases rather than systematic detection.

When comparing panel sequencing to WES, the panel detected tumor-informed SNVs in 55% of patients, outperforming WES, which detected SNVs in 33% (Fig. 5B). The increase in SNV detection was particularly notable in brain tumors (Panel: 42%, WES: 11%) and sarcomas (Panel: 100%, WES: 71%). For other malignancies, WES slightly outperformed the panel (Panel: 43%, WES: 57%, Fig. 5B). We also compared ctDNA fraction and VAF between WES and panel sequencing (Additional file 1: Fig. S7C–F). The WES mVAF exhibited a weak correlation with the lcWGS-based ctDNA fraction (*r* = 0.57, Additional file 1: Fig. S7C), likely due to subclonal SNVs. This correlation improved (*r* = 0.67) when using the maximal VAF instead (Additional file 1: Fig. S7E). Panel sequencing had a stronger correlation with lcWGS ctDNA fraction (*r* = 0.84 for mVAF and *r* = 0.83 for maximal VAF) (Additional file 1: Fig. S7D and Fig. S7F). To further benchmark CES against established ctDNA metrics, we performed receiver ROC analyses (Fig. 5C). CES outperformed both the short-to-long fragment ratio and ichorCNA tumor fraction in discriminating samples with independently confirmed ctDNA. Using a composite orthogonal ground truth (ichorCNA TF ≥ 3%, WES mVAF > 0, or panel mVAF > 0), CES achieved an AUC of 0.74 compared with 0.67 for the short-to-long ratio. To avoid circularity resulting from shared copy-number information between CES and ichorCNA, we additionally evaluated both metrics against a VAF-only ground truth. Under this criterion, CES maintained superior performance (AUC = 0.65) relative to ichorCNA tumor fraction (AUC = 0.52). Correlation analyses (Fig. 5D) showed moderate associations between CES and panel-derived VAF (ρ = 0.55) as well as ichorCNA tumor fraction (ρ = 0.42), whereas the correlation with WES-derived VAF was weak (ρ = 0.21). These findings suggest that CES captures complementary cfDNA features beyond those measured by conventional ctDNA quantification approaches. Importantly, additional analyses demonstrated that cfDNA performance was largely independent of pre-analytical variables. Neither cfDNA input nor cfDNA purity showed meaningful correlations with ctDNA fraction, short-to-long ratio or CES (Additional file 1: Fig. S8A–E), and no association between patient age and CES was observed (Additional file 1: Fig. S8F). Similarly, only weak or non-significant relationships were detected between cfDNA purity and sequencing-derived VAF metrics in WES and targeted panel sequencing (Additional file 1: Fig. S8G–H), supporting the robustness of applied cfDNA sequencing platforms across varying sample quality and input conditions. Sample-matched analyses (*n* = 29) revealed good concordance across sequencing methods (Fig. 5E). lcWGS was most effective for identifying high-priority CNVs, whereas panel sequencing showed the strongest performance for detecting very high-priority SNVs, followed by WES for detecting high-priority SNV targets (Fig. 5F and Additional file 1: Fig. S7G). The selection of sequencing approach should be tailored to the specific diagnosis, liquid biopsy purpose (such as MRD vs. target detection), mutation types, and cfDNA yield.

### Liquid biopsy-based detection of temporal heterogeneity

**Fig. 6:**
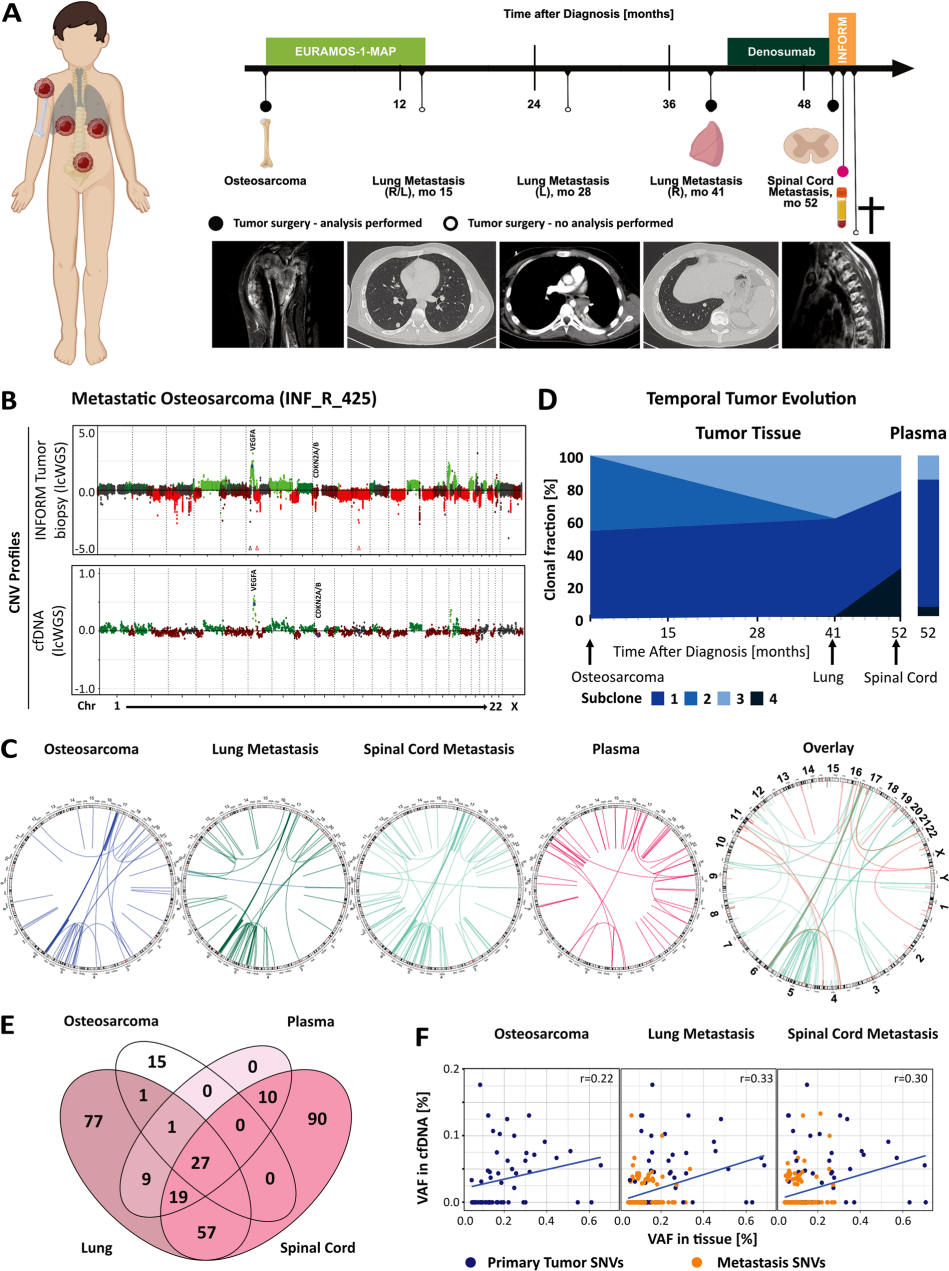
Detection of Temporal Tumor Heterogeneity in Liquid Biopsy Samples. **A** Graphical summary of tumor sites diagnosed over time for an individual INFORM patient with metastatic osteosarcoma. Black dots indicate tumor episodes with available molecular tumor data, black circles indicate tumor episodes without available molecular tumor data. **B** WGS-derived CNV profiles of a tumor tissue biopsy (upper) and of a plasma-derived cfDNA sample obtained following surgery analyzed with in silico short fragment filtering for improved tumor detection (lower). Both *VEGFA* (chromosome 6p) and *CDKN2A/B* (chromosome 9p) amplifications that were reported by the molecular INFORM tumor board for the tissue biopsy (52 months after diagnosis) were also detected in a simultaneously obtained plasma sample. **C** Circos plots of structural variants per indicated site and as overlay plot of the primary osteosarcoma (blue), a lung metastasis (41 months after diagnosis, green), a spinal cord metastasis (52 months after diagnosis, turquois) and plasma cfDNA (52 months after diagnosis, red). **D** Temporal evolution analysis of tumor tissues biopsies from indicated time points based on WES-derived VAF of SNVs. Subclonal assignment to the cfDNA sample revealed a major subclone already present at primary diagnosis and two minor subclones originating from subsequent lung and spinal cord metastatic sites. **E** Venn diagram of somatic SNVs revealing that the liquid biopsy analysis reflects clonal contribution from the different tumor sites. **F** Evolution of tumor SNVs and their respective VAF in tumor tissue (primary/metastasis, WES) and plasma-derived cfDNA (lcWGS). Correlation plot of VAF in cfDNA and tissue of indicated tumor sites. Dark blue indicates SNVs detected in the primary osteosarcoma, orange indicates SNVs that evolved over time (*r* = 0.22 (Osteosarcoma), *r* = 0.33 (Lung Metastasis), *r* = 0.33 (Spinal Cord Metastasis)

**Fig. 7:**
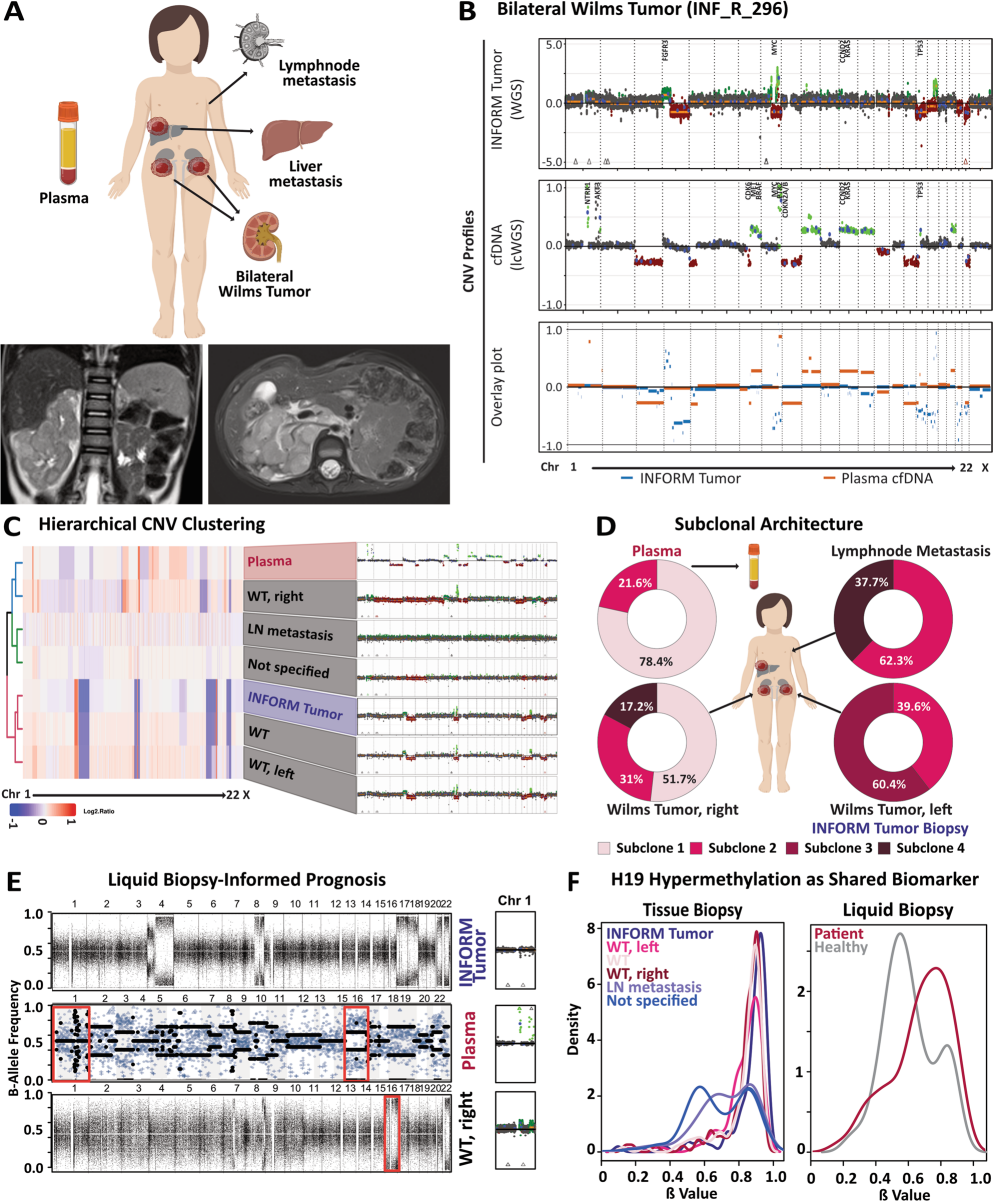
Liquid biopsy-based Detection of Spatial Tumor Heterogeneity. **A** Graphical summary of tumor diagnosis at different locations for an individual INFORM patient with bilateral, metastatic Wilms tumor (WT). MRI of the indicated patient highlighting the liver metastasis. **B** Genome-wide CNV profiles derived from WGS of tumor tissue biopsy (upper), cfDNA lcWGS (middle) and overlay plot of tumor (blue) and plasma (orange, lower). Colors of the CNV profile represent neutral CNV state (grey), deletions (red), gains (3 N, green) and focal amplifications (> 3 N, light green). **C** Unsupervised hierarchical clustering of CNVs by Pearson’s correlation identified the WT from the right site to be the predominant plasma clone. There is a discordance of the cfDNA and the WT sample analyzed for the INFORM molecular tumor board. Corresponding CNV profiles are highlighted on the right. **D** Subclonal architecture within tumors from indicated sites and plasma cfDNA based on WES-derived VAF of SNVs. Subclonal assignment to the cfDNA sample revealed a major subclone (Subclone 1, 78.4%) unique for WT from the right site (Subclone 1, 51.7%), which is not present in the WT sample analyzed for the INFORM molecular tumor board (biopsy from the left side). **E** B-allele frequency plot showed no loss-of-heterozygosity (LOH) of chromosomes 1q or 16p in the INFORM tumor tissue analysis. In contrast, the plasma sample presented partial LOH on chromosome 1 and a distinct LOH on the entire chromosome 16 (highlighted in red), stratifying patient a poor prognosis. **F** ß-Value density plot of the H19 locus revealed H19 hypermethylation as shared early oncogenic hit of all investigated tumor tissue biopsies. In plasma liquid biopsies, H19 methylation patterns allowed a clear discrimination of the WT patient from healthy controls

Liquid biopsies can reflect genetic alterations across different stages of a tumor, aiding therapeutic target identification and disease monitoring. By analyzing serial liquid biopsies from primary diagnosis, relapse, and metastasis, we assessed temporal tumor evolution (Fig. 6) and spatial heterogeneity (Fig. 7). Patient INF_R_425, diagnosed with osteosarcoma of the right humerus, underwent surgery and chemotherapy, followed by metastases and spinal lesions. The occurrence of a spinal lesion triggered enrollment in the INFORM registry at month 52 after diagnosis, with a liquid biopsy plasma sample being available alongside material from the primary tumor and a lung metastasis (41 months post-diagnosis) for parallel molecular analysis (Fig. 6A). lcWGS CNV analysis of the plasma sample revealed chromosomal instability, with *VEGFA* amplification and *CDKN2A/B* deletion present in the primary tumor and spinal lesion, but only *VEGFA* amplification in the lung metastasis (Fig. 6B and Additional file 1: Fig. S9A–B). Comparison of structural rearrangements in Circos plots highlighted distinct genomic instability patterns at each sampling site (Fig. 6C and Additional file 1: Fig. S9C). To investigate tumor evolution, we inferred clonal hierarchies using VAF-based SNV clustering from all tumor tissues and plasma (Additional file 1: Fig. S9D). The primary tumor had four subclonal lineages, three of which were detectable in the plasma (Fig. 6D). Subclone 1 (54%) was stable across metastasis, and predominated in the cfDNA (77%), while subclone 2 (46%) became extinct. Metastatic subclones 3 and 4 from the lung (39%, months 41) and spinal metastases (31%, months 52) were both detected in plasma (15% and 8%, respectively). Subclone 1 remained predominant, with cfDNA composition closely resembling the spinal lesion (Fig. 6D).

We also assessed the impact of sequencing method and mean depth of coverage on SNV detection and subclonal composition in plasma cfDNA using low (1x) and medium (16x) mean depth of coverage WGS, WES, and panel sequencing (Additional file 1: Fig. S9E–H). lcWGS detected shared and unique CNVs, and WGS/WES showed positive correlation between SNV amounts and mean depth of genomic coverage (Additional file 1: Fig. S9E–G), while the panel covered only a single SNV (Additional file 1: Fig. S9H). We examined mutation representation over time across tumor sites: primary osteosarcoma (64%, diagnosis), lung metastasis (23%, month 41), and spinal cord metastasis (28%, month 52) in cfDNA (Fig. 6E). Plasma-based detection of unique SNVs further supported the resemblance of cfDNA to the spinal lesion, with 10 unique SNVs from the spinal metastasis, 1 from the lung, and none from the primary tumor (Fig. 6E). VAF-based correlation analyses showed that as the disease progressed, the VAF correlation between liquid biopsy and tumor sites increased, indicating that plasma-based liquid biopsy effectively reflected the evolving subclonal architecture (Fig. 6F and Additional file 1: Fig. S9I). These results suggest that temporal tumor evolution can be monitored via cfDNA analysis.

### Liquid biopsy captures spatial tumor heterogeneity, enabling location-independent molecular profiling

Performance comparisons revealed high plasma tumor content but low concordance with tissue SNVs and CNVs for Patient 25 (INF_R_296), diagnosed with metastatic bilateral Wilms tumor progressing under first-line SIOP 2001 GPOH therapy (HR, Stage V, Fig. 7A). At INFORM enrollment, metastases were present in the liver, lymph nodes, and abdominal wall (Fig. 7A). The INFORM pipeline detected *MYC* amplification, a *TP53* mutation, and *KDM1A* overexpression in a Wilms tumor sample (= INFORM tumor), for which no specific anatomical location was available. The *MYC* amplification is also evident in the CNV profiles (Fig. 7B and Additional file 1: Fig. S10A). For the liquid biopsy at enrollment, ichorCNA reported 37.4% ctDNA with distinct alterations, including focal amplifications on chromosome 1 and whole chromosome gains of 10, 12, and 13 (Fig. 7B). CNV analysis showed overlapping but also unique aberrations between tumor and plasma (Fig. 7B). Genotyping confirmed sample identity (Additional file 1: Fig. S10B). Tracing cfDNA origins, WGS and methylation analysis of primary tumors from both kidneys, an unspecified site, and lymph node metastasis revealed that the INFORM tumor corresponded to the left Wilms tumor, while the right tumor was the predominant cfDNA source (Fig. 7C). Correlation analysis of CNV profiles revealed a strong similarity between cfDNA and the right-sided tumor (Pearson *r* = 0.7), whereas no meaningful correlation was observed with the left-sided tumor (*r* = 0.04) (Additional file 1: Fig. S10C). Consistent with these findings, methylation analysis demonstrated the highest similarity between plasma cfDNA and the right-sided Wilms tumor sample (Additional file 1: Fig. S10D–E ).

SNV-based phylogenetic analysis identified two dominant subclones in cfDNA (78% and 22%), with the latter shared across all tumor sites (Fig. 7D and Additional file 1: Fig. S10F). However, the most prominent INFORM tumor subclone was absent from cfDNA, highlighting site-specific divergence (Fig. 7D and Additional file 1: Fig. S10G). Notably, three gene alterations were cfDNA-exclusive, while two were unique to the INFORM tumor (Additional file 5: Table S3). These findings underscore the need for further validation of liquid biopsy-detected targets for clinical actionability.

Furthermore, we investigated prognostic Wilms tumor biomarkers with potential relevance for therapy intensification [50, 51]. Loss of heterozygosity (LOH) on 1p/16q was detected in plasma but not in the INFORM tumor, confirming its right Wilms tumor origin (Fig. 7E, left). The prognostically relevant 1q gain appeared in cfDNA as a focal amplification (up to 10 copies) and was traced to the right tumor, where it spanned a broader region (Fig. 7E, right). To identify a shared Wilms tumor biomarker, we analyzed *H19* locus methylation across tumor sites [33]. All tumors with recognizable content showed clear *H19* hypermethylation, while the lymph node metastasis and a low-content sample exhibited an indistinct ß-value distribution (Fig. 7F, left). Notably, *H19* hypermethylation was also detected in cfDNA, distinguishing tumors from normal controls (Fig. 7F, right).

These findings illustrate how liquid biopsies capture regionalized risk factors undetected by single-tumor analysis, potentially informing (neo)adjuvant strategies. *H19* hypermethylation may serve as a universal Wilms tumor biomarker, warranting further investigation for clinical implementation.

## Discussion

The INFORM registry has demonstrated the feasibility of comprehensive molecular profiling for pediatric tumor tissues in a real-world, multi-institutional setting [7]. However, high-level evidence targets for matched therapies that improve clinical outcomes remain limited (~ 10%) and cannot be serially monitored via invasive biopsies [7, 8].

To address this, pediatric precision oncology programs are integrating drug sensitivity profiling [52], proteomics, and liquid biopsy to capture spatial and temporal tumor heterogeneity. Here, we present a comparative plasma-based DNA sequencing approach for tumor detection, molecular profiling, and target identification in high-risk pediatric brain and solid tumors. In line with previous studies, cfDNA levels were higher in patients with certain solid tumors, such as neuroblastoma [4]. Although brain tumors represent the largest diagnostic group and plasma cfDNA is typically considered an unreliable liquid biopsy source, our combined analytical approach successfully detected tumors in the majority of patients. With a 93% detection rate, our method demonstrates the potential of liquid biopsy for molecular residual tumor assessment, even in challenging cases. Our study confirmed WES superiority for target detection, consistent with the MAPPYACTS study [4]. At the same time, variability in preanalytical conditions, including cfDNA input and purity, remains an inherent challenge in real-world precision oncology settings. These factors can influence analytical sensitivity and should be carefully considered when interpreting results. In particular, parameters such as cfDNA input, tumor fraction, and sequencing depth define the limits of detection and may affect the robustness of downstream analyses. Accordingly, while optimized workflows [19] can mitigate some of these constraints, a nuanced interpretation of liquid biopsy data that accounts for these sensitivities is essential for clinical application [20, 46, 47]. A further limitation is the lack of standardized reference materials that recapitulate cfDNA fragmentation patterns and complex genomic alterations, which currently hampers the precise definition of analytical limits of detection across platforms. In this context, the identification of cfDNA-exclusive mutations, as observed in our cohort, highlights the potential of liquid biopsy to capture spatial and temporal tumor heterogeneity, particularly under treatment pressure, but also raises important questions regarding their clinical actionability. However, their clinical interpretation requires careful consideration. While technical advances, including UMI-based error correction, reduce the likelihood of artefactual variant calls, plasma-exclusive findings, especially those at low VAF, should ideally be confirmed through repeated sampling and orthogonal validation methods (e.g., ddPCR or targeted deep sequencing) before informing clinical decisions. From a regulatory and clinical perspective, current frameworks, including ESMO recommendations [53] for ctDNA testing and Carelon clinical appropriateness guidelines, generally prioritize tissue-confirmed alterations for therapy selection, and the actionability of cfDNA-derived variants without tissue correlation remains limited outside controlled clinical trial settings. In this context, current guidelines also emphasize the importance of validated assay sensitivity, clearly defined input parameters, and established limits of detection, particularly for low-frequency variants. While the high-depth targeted sequencing approach used in our study aligns with these requirements for sensitive variant detection, further prospective studies are required to establish its clinical utility across diverse pediatric tumor entities and clinical scenarios.

Recurrent alterations in genes such as *PIK3CA*, identified as one of the most frequently mutated genes in our cohort, are of particular interest. Activation of the PI3K/AKT pathway has been associated with tumor progression, therapy resistance, and relapse across multiple cancer types, including rhabdomyosarcoma [54]. Moreover, crosstalk between the PI3K/AKT and MAPK pathways has been described, suggesting that tumors harboring *PIK3CA* alterations may benefit from combinatorial or alternative targeting strategies involving MAPK pathway inhibition [55]. Notably, the MAPK pathway represents one of the most commonly implicated pathways in both relapsed disease and targeted treatment approaches, further underscoring the relevance of pathway-level interpretation of cfDNA findings. In this context, integrative approaches combining cfDNA profiling with orthogonal data sources, such as drug sensitivity assays [56] or proteogenomic analyses, may help refine prioritization strategies. Ultimately, prospective clinical studies are required to define evidence thresholds and establish guidelines for the implementation of plasma-derived alterations in precision oncology decision-making.

Liquid biopsy-based molecular residual disease detection strongly predicts recurrence in adult cancers and may guide treatment decisions [57–63]. However, its clinical utility in pediatric oncology remains under investigation. Our study, focused on high-risk patients, achieved a 93% tumor detection rate by integrating in silico DNA size selection and segmental copy number analysis within the CES algorithm. Notably, CES identified tumor progression months before clinical detection, consistent with observations from adult liquid biopsy studies [63, 64]. Nevertheless, the limited availability of age-matched healthy controls, potential class imbalance, and the presence of non-oncological pathological conditions, e.g., cancer predisposition syndromes or vascular and autoimmune diseases [65, 66], should be considered, as these factors may influence performance metrics, particularly predictive values (e.g., PPV and NPV). While cross-validation provides an important internal assessment of model performance, independent external validation in separate patient cohorts will be required to confirm the generalizability and robustness of the classification model and its predefined decision thresholds. Future studies with larger, well-matched control cohorts and prospective validation in first-line settings will be essential to further define and strengthen the clinical utility of CES for prognostication and response monitoring [67].

Pediatric cancers exhibit genetic heterogeneity between primary and metastatic sites [68–71]. In exemplary cases, plasma-exclusive findings were mapped to previously unsampled lesions, highlighting the power of minimally invasive technologies to capture clinically relevant subclones for targeted therapy and risk stratification. Our data support an integrated liquid biopsy workflow combining cfDNA screening via lcWGS with alteration-adapted analysis, complementing molecular tumor profiling within precision oncology programs like INFORM. The selection of sequencing approaches, data interpretation, and quality measures necessitates clinical decision support tools for streamlined translation into clinical practice.

## Conclusions

Our pan-cancer liquid biopsy study demonstrated the effectiveness of three NGS technologies in an orthogonal comparison, achieving a 92% technical success rate despite small sample volumes and low genetic redundancy. By integrating in silico DNA size selection and segmental copy number analysis within the CES algorithm, our approach achieved a 93% tumor detection rate, highlighting the potential of liquid biopsy for molecular residual tumor assessment, even in challenging cases. WES proved to be the most effective approach for target detection. In addition, CES enabled the detection of tumor progression months before clinical detection, supporting its potential for disease monitoring. Our pipeline is now integrated into INFORM as an additional molecular layer. Ongoing prospective applications within early-phase and therapy optimization trials will further define the clinical impact of liquid biopsy in pediatric oncology [7, 72].

## Supplementary information

Additional file 1: Supplementary Figures

Additional file 2: Supplementary Methods

Additional file 3: Table S1. Cohort description

Additional file 4: Table S2. Clinically relevant genes

Additional file 5: Table S3. Wilms-specific alterations

## Data Availability

The high-throughput sequencing datasets generated during this study are available in the European Genome-phenome Archive (EGA) under accession number EGAS50000000393 ( https://ega-archive.org/studies/EGAS50000000393) [73]. Additional pediatric control data were previously published and are available via EGAD00001007080 (https://www.ega-archive.org/datasets/EGAD00001007080) [21]. All NGS data were processed using standardized workflows provided by the DKFZ Omics IT and Data Management Core Facility (ODCF) where all genomics annotation information were hosted [40].

## Abbreviations

CES: ctDNA Estimation Score
cfDNA: Cell–free DNA
ctDNA: Circulating tumor DNA
CNV: Copy number variation
SNV: Single nucleotide variant
Indel: Insertion and deletion
lcWGS: ow–coverage whole–genome sequencing
WGS: Whole–genome sequencing
WES: Whole–exome sequencing
NGS: Next–generation sequencing
VAF: Variant allele frequency
UMI: Unique molecular identifier
SLRatio: Short–to–long fragment ratio
PoN: Panel of normals
CBS: Circular binary segmentation
ROC: Receiver operating characteristic
AUC: Area under the curve
LOOCV: Leave–one–out cross–validation
MRD: Minimal residual disease
PFS: Progression–free survival
MRI: Magnetic resonance imaging
CNV: Copy number variation
SV: Structural variant
mVAF: Median variant allele frequency
MAD: Median absolute deviation
GC: Guanine–cytosine (content)
LOESS: Locally estimated scatterplot smoothing
EPIC: Infinium methylation EPIC array
DKFZ: German cancer research center
DKTK: German cancer consortium
NCT: National center for tumor diseases
INFORM: Individualized therapy for relapsed malignancies in childhood
GPOH: German society for pediatric oncology and hematology
EGA: European Genome–phenome Archive
