# Supplementary material for: Circulating tumor DNA precision oncology enables effective and sensitive molecular diagnostics and actionable target detection in pediatric solid tumors - the INFORM experience
Source: Genome Med. 2026 Jul 27;18:112. doi: 10.1186/s13073-026-01737-4 (PMC13411563; doi:10.1186/s13073-026-01737-4)
Supplement: Supplementary file 1 — Additional file 1: Supplementary Figures [file 13073_2026_1737_MOESM1_ESM.docx]

Additional file 1

**
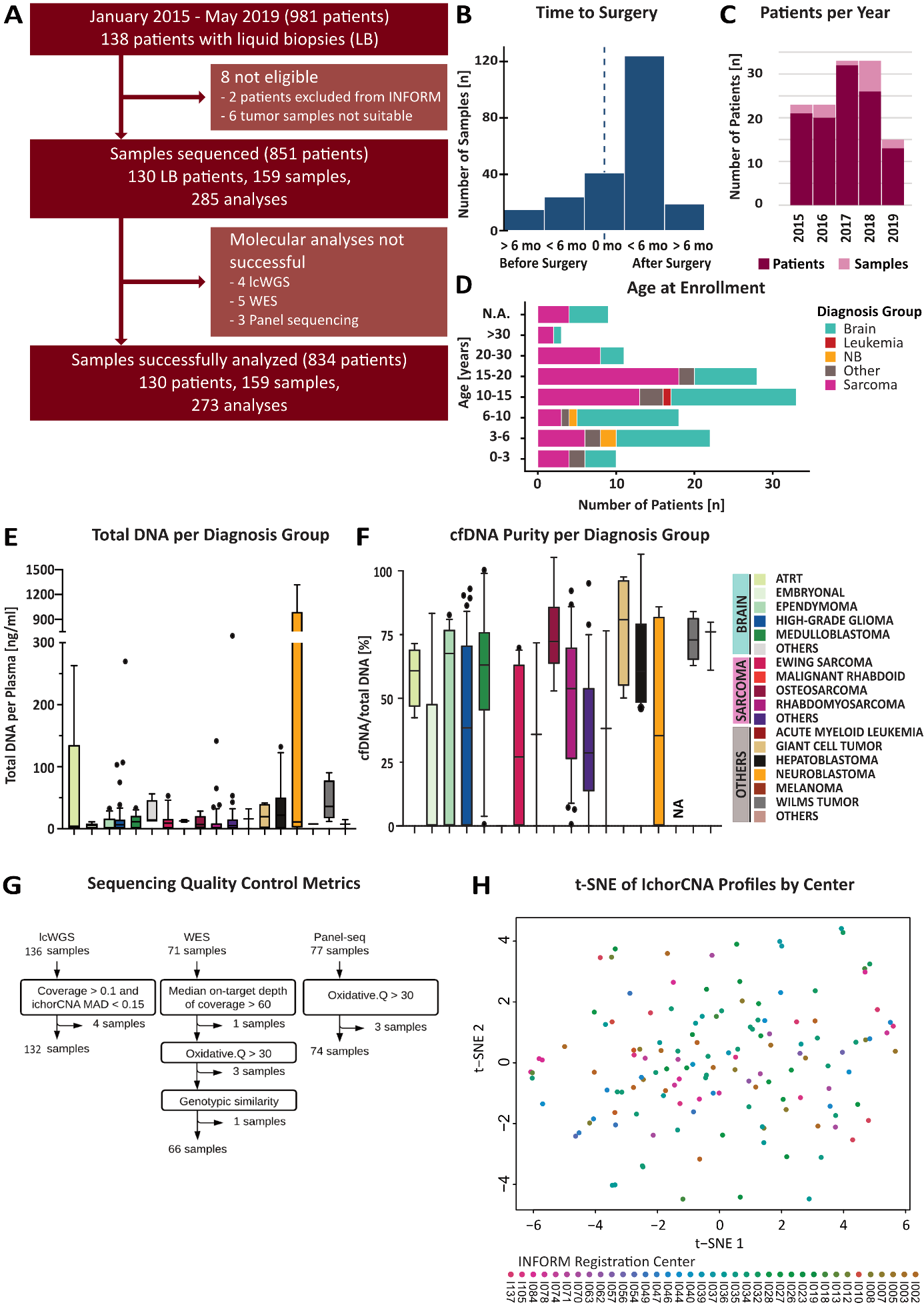
Fig. S1** **Cohort Characteristics and Quality Metrics A)** Cohort overview of INFORM patients registered between January 2015 and May 2019 with liquid biopsy blood plasma material available. **B)** Distribution of time between surgery and plasma collection. Samples are grouped in intervals before and after surgery, with the dashed line indicating the time of surgery (0 mo.). **C)** INFORM patient registrations with available liquid biopsy material over time (dark red - patients, light red - liquid biopsy samples). **D)** Age distribution of patients’ enrollment stratified by diagnosis group. Bars show the number of patients per age category, with colors indicating tumor types (brain, leukemia, neuroblastoma (NB9, other, and sarcoma). **E)** Total amounts of DNA isolated per ml plasma of the indicated diagnosis groups. ­ **F)** Purity of isolated cell-free DNA (cfDNA) calculated as the ratio of cfDNA to total DNA in %, determined by Bioanalyzer fragment length measurements. **G)** Quality control metrics for low-coverage whole-genome sequencing (lcWGS), whole-exome sequencing (WES) and panel sequencing data revealing the final liquid biopsy cohort for orthogonal sequencing comparison of 159 samples being subjected to 273 analyses. Of all 136 lcWGS samples, 4 samples were excluded due to mean depth of genomic coverage of lower than 0.1x or ichorCNA median absolute deviation (MAD) less than 0.15. Of all 71 WES samples, 1 sample was excluded due to an on-target mean depth of coverage lower than 60x and 3 samples were excluded due to an excessive level of oxidative artefacts. Of all 77 panel sequencing samples, 3 had to be excluded due to high oxidative artefact rates. MAD – median absolute deviation as QC output of ichorCNA. **H)** t-SNE projection of ichorCNA profiles across all cfDNA samples, colored by collection center. No distinct clustering by center is observed, indicating the absence of center-specific batch effects and suggesting that variation is primarily driven by biological differences rather than technical artifacts.


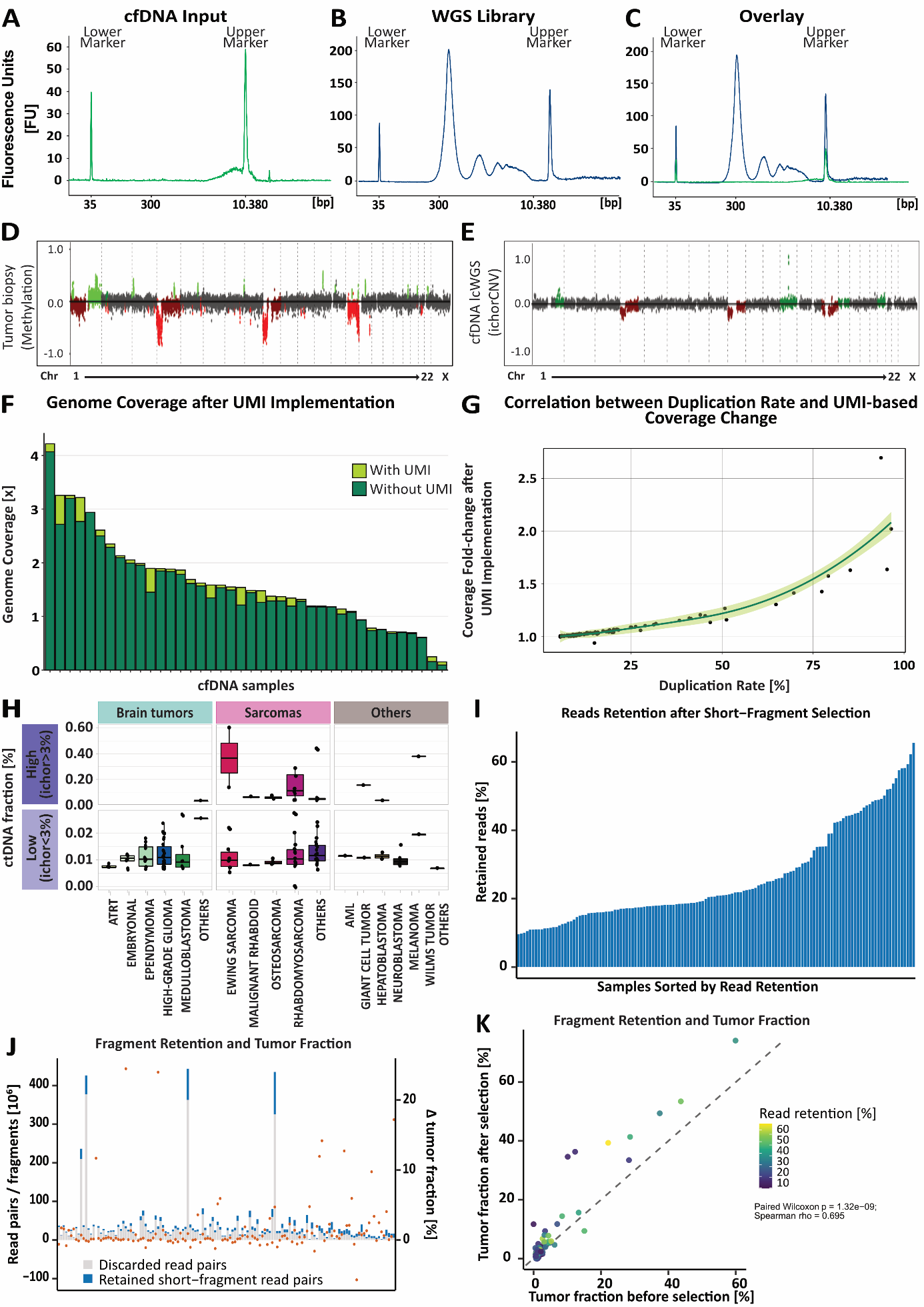


**Fig. S2 Increased Mean Depth of Genome Coverage of Low-coverage Whole-Genome Sequencing (lcWGS) through Implementation of Unique Molecular Identifier (UMI)** **A)** Bioanalyzer size profile for cell-free DNA (cfDNA) isolated from plasma of a patient with alveolar rhabdomyosarcoma and **B)** the respective library for lcWGS. **C)** Overlay plot of cfDNA and library size profiles illustrates enrichment of DNA by a library protocol adapted for low-input material. The lower (35 bp) and upper (10.380 bp) markers are indicated to facilitate interpretation of yield and fragment size distribution. **D)** EPIC array-derived copy number variation (CNV) profile of tumor DNA and **E)** corresponding lcWGS-based CNV profile (library shown in B) from plasma cfDNA for patient in A. This exemplary case illustrates the feasibility of lcWGS-based CNV detection from minimal cfDNA inputs. **F)** Implementation of UMI-based deduplication (light green) improved mean depth of genome coverage over MarkDuplication (dark green) by on average 7% in lcWGS samples. **G)** Increasing mean depth of coverage in lcWGS showed positive correlation with the degree of duplicated reads removed by MarkDuplication. **H)** Calculated ctDNA fractions for indicated tumor entities plotted for high (ichor>3%, dark purple) and low (ichor<3%, light purple) ctDNA fraction groups. Data revealed high ctDNA fractions for Ewing sarcomas, rhabdomyosarcomas, and other sarcomas as well as for Wilms tumors while brain tumors and other solid tumors mostly showed low ctDNA fractions. **I)** Fraction of reads retained after short-fragment selection. Bar plot showing the percentage of sequencing reads retained after *in silico* short-fragment selection across all samples. Samples are ordered by increasing read retention. **J)** Stacked bar plot showing the total number of sequencing read pairs per sample, partitioned into retained short-fragment read pairs (blue) and discarded read pairs (grey). Samples are ordered by increasing fraction of retained short fragments. The orange dots indicate the change in ctDNA fraction (Δ ctDNA fraction [%]) after short-fragment selection for each sample. **K)** Scatter plot comparing ctDNA fraction estimates before and after short-fragment selection across samples. Each point represents a sample and is colored according to the fraction of reads retained after short-fragment selection. The dashed line indicates the line of identity. Short-fragment

selection leads to a significant increase in estimated tumor fraction (paired Wilcoxon test p = 1.32 × 10⁻⁹), with a strong positive correlation between pre- and post-selection values (Spearman ρ = 0.695).


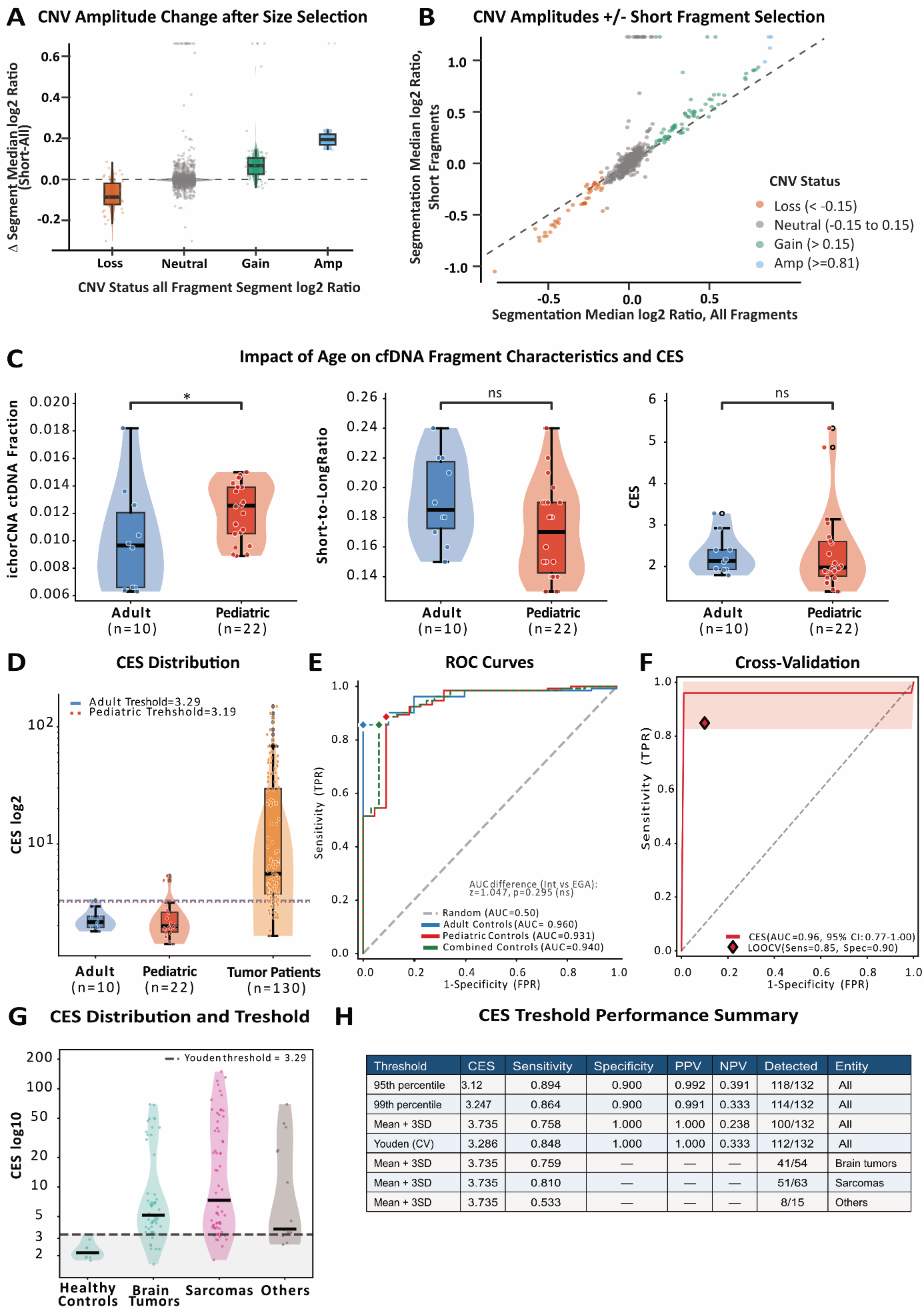


**Fig. S3 CES Benchmarking A)** Distribution of changes in segment median log2 ratios (Δ log2 ratio) after short-fragment selection, stratified by CNV status (loss, neutral, gain, amplification) as defined in the entire dataset. Each point represents a segment, with boxplots summarizing the distribution per category. Short-fragment selection leads to a decrease in amplitude for loss segments and an increase for gain and amplified segments, indicating enhanced CNV signal separation. **B)** Scatter plot comparing segment median log2 ratios before (all fragments) and after short-fragment selection. Each point represents a segment and is colored by CNV status. The dashed line indicates the line of identity. Deviations from the diagonal reflect CNV amplitude shifts following short-fragment enrichment. **C)** Comparison of ichorCNA-derived ctDNA fraction (left), short-to-long fragment ratio (middle), and CES (right) between adult (n = 10) and pediatric (n = 22) healthy control samples. Each point represents an individual sample, with violin plots and boxplots indicating the distribution within each group. Pediatric samples show a significantly higher ctDNA fraction compared to adults (*p < 0.05), while no significant differences (ns) are observed for the short-to-long fragment ratio or CES. **D)** Distribution of CES across adult controls (n = 10), pediatric controls (n = 22), and tumor patients (n = 130). Each point represents an individual sample, with violin plots and boxplots summarizing group distributions. Dashed lines indicate the optimal CES thresholds for adult (blue, 3.29) and pediatric (red, 3.19) controls. Tumor samples show markedly higher CES values compared to controls. **E)** Receiver operating characteristic (ROC) curves evaluating the performance of CES for distinguishing tumor patients from controls. Curves are shown for adult controls (blue), pediatric controls (red), and combined controls (green), with corresponding area under the curve (AUC) values of 0.960, 0.931, and 0.940, respectively. The dashed diagonal line indicates random classification (AUC = 0.50). No significant difference in AUC is observed between control groups (ΔAUC = 1.047, p = 0.295). **F)** Mean receiver operating characteristic (ROC) curve from repeated stratified 10-fold cross-validation (100 repeats) for CES classification of cancer patients (n = 132) versus healthy controls (n = 32). The shaded area represents the 95% confidence band. The diamond indicates the operating point from leave-one-out cross-validation (LOOCV). **G)** Distribution of CES scores across healthy controls, brain tumors, sarcomas, and other malignancies. Individual data points are shown with jitter; horizontal bars indicate group medians. The dashed line denotes the youden optimal threshold derived from healthy controls (CES = 3.29). The shaded grey region represents the healthy reference range. **H)** Summary table of CES classification performance at candidate detection thresholds, optimal CES threshold, AUC, sensitivity, specificity, PPV, NPV and F1 score.


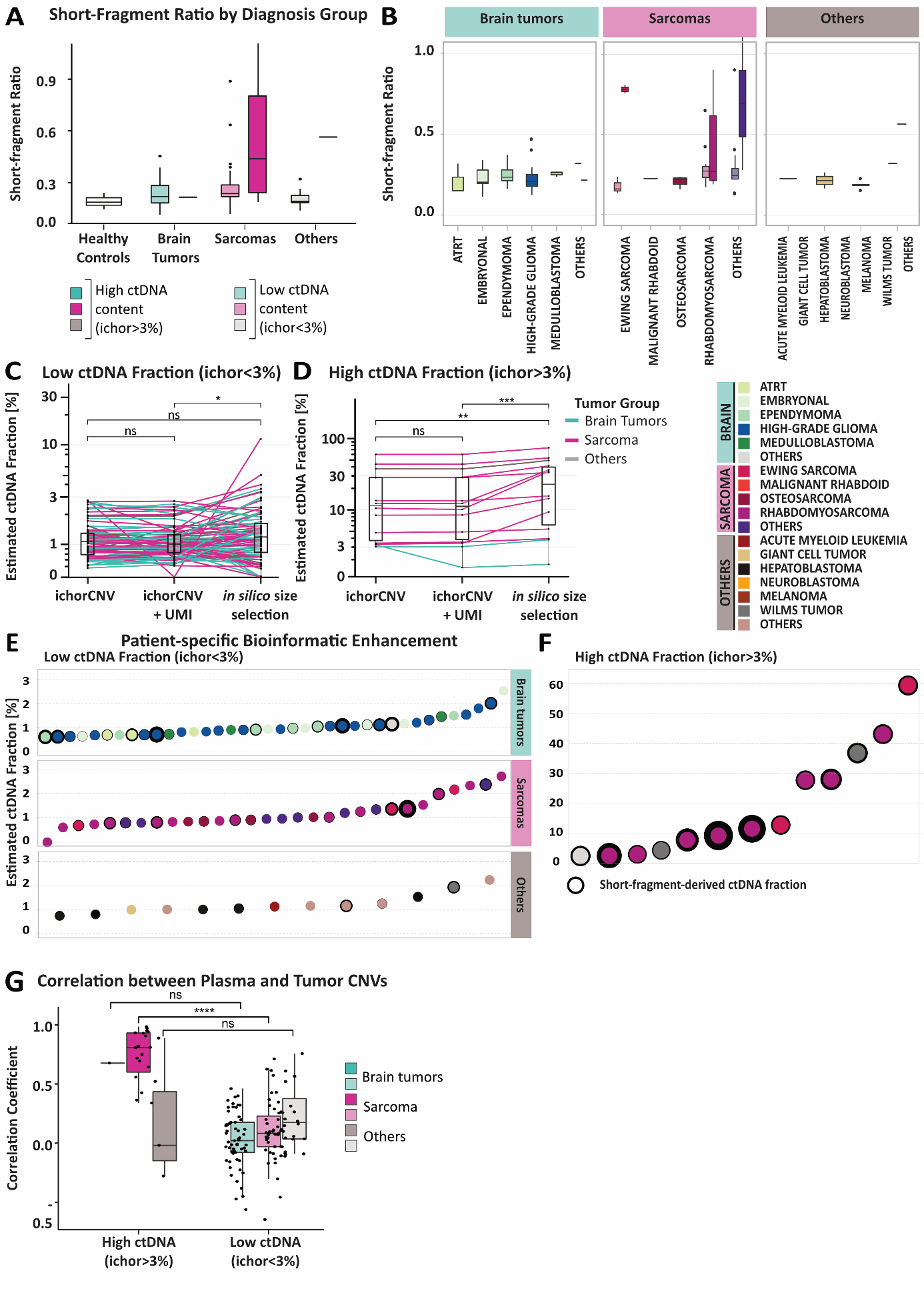


**Fig. S4 Low-Coverage Whole-Genome Sequencing (LcWGS) of Liquid Biopsies Recapitulated Tumor Copy Number Variations (CNVs) with High Sensitivity and Specificity A)** Box plot representation of short-fragment ratio in plasma samples of healthy controls, brain tumor, sarcoma, and patients with other tumors showing strong enrichment of short cell-free DNA (cfDNA) fragments in sarcoma patients. **B)** Distribution of short-fragment ratios for indicated tumor entities revealing high enrichment of short fragments in Ewing sarcomas, Rhabdomyosarcomas and other sarcomas. **C-D)** Estimated circulating tumor DNA (ctDNA) fraction based on ichorCNA for **C)** low (ichor<3%) and **D)** high (ichor> 3%) ctDNA samples. Plasma ctDNA fraction was slightly improved bioinformatically by implementing unique molecular identifier (UMIs) but significant higher ctDNA fractions could be extracted by performing in silico short fragment size selection. P-values were calculated by paired Wilcoxon signed rank test with *= p<0.05** = p<0.01, *** = p<0.001. **E-F)** Increased mean depth of coverage by UMI implementation resulted in improved ctDNA fraction detection shown for individual patients. **E)** Tumor detection in plasma samples of low ctDNA patient group (ichor<3% ctDNA fraction) revealed strongest improvement for brain tumors (upper graph), followed by sarcomas (middle graph), and other tumors. IchorCNA estimated ctDNA fraction is plotted on the y-axis and the increase of the estimated ctDNA fraction following in silico size selection is indicated by black circles drawn to scale. **F)** Tumor detection in plasma samples of high ctDNA patient group (≥3% ctDNA fraction) revealed stronger improvement in ctDNA fraction estimation for sarcomas over other tumors. Tumor entities are indicated with color code shown in legend above. **G)** Pearson correlation coefficient of genomic log2 ratio as surrogate marker for copy number variation (CNV) similarities between tumor and matched cfDNA samples showed higher correlation for high (ichor>3%) than for low (ichor<3%) ctDNA groups irrespective of diagnosis groups.


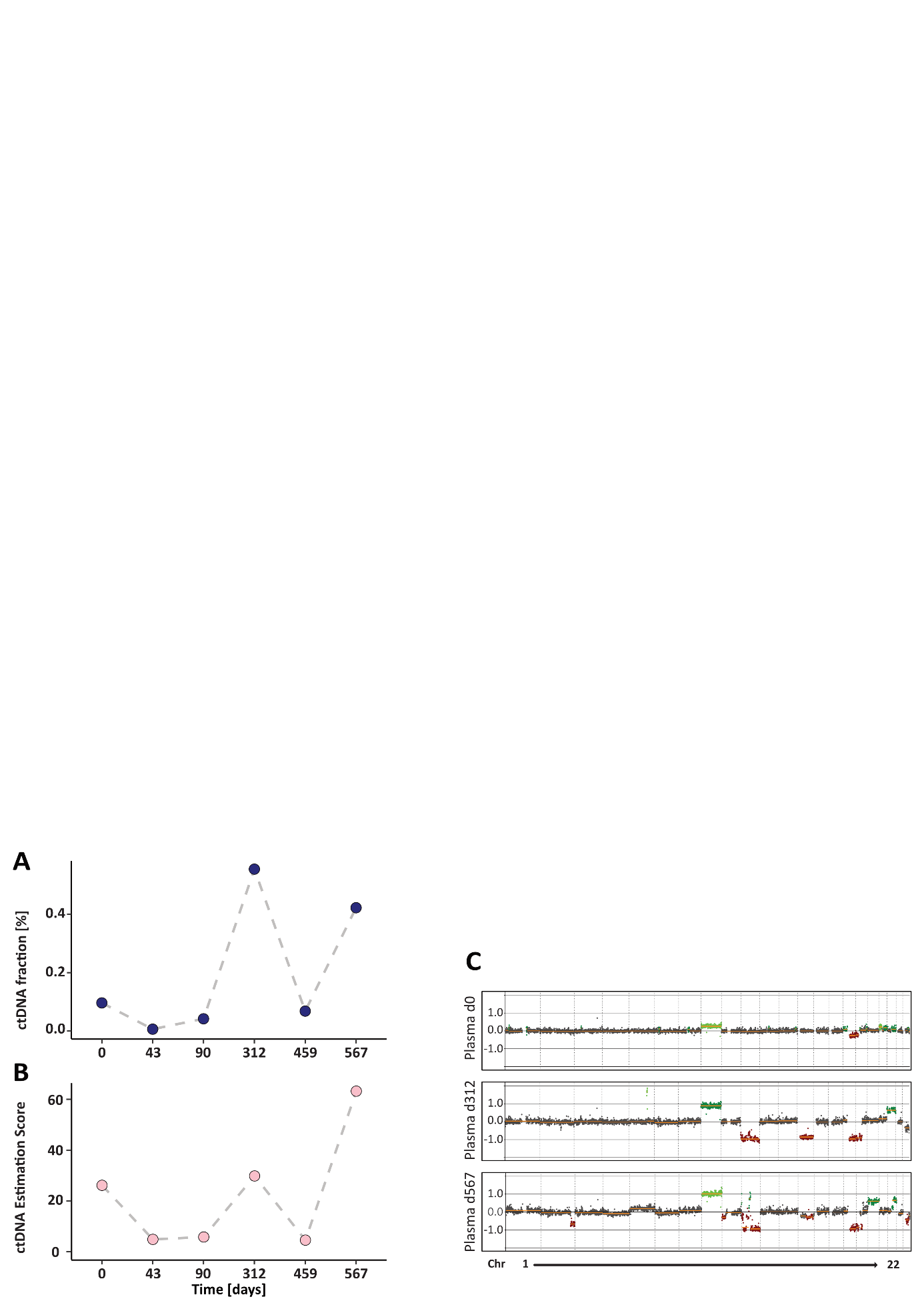


**Fig. S5 CES Validation in Independent Ewing Sarcoma Cohort^25^ A)** IchorCNA estimated ctDNA fraction and **B)** ctDNA Estimation Score (CES) plotted over clinical course of Ewing sarcoma patient. Clinical events include diagnosis (day 0), first relapse (day 312), and second relapse (day 567) are annotated. Both metrics increase in concordance with relapse events, supporting their utility as non-invasive biomarkers for disease burden and progression. **C)** Genome-wide CNV profiles derived from cfDNA WGS are depicted at diagnosis (day 0), first relapse (day 312), and second relapse (day 567).


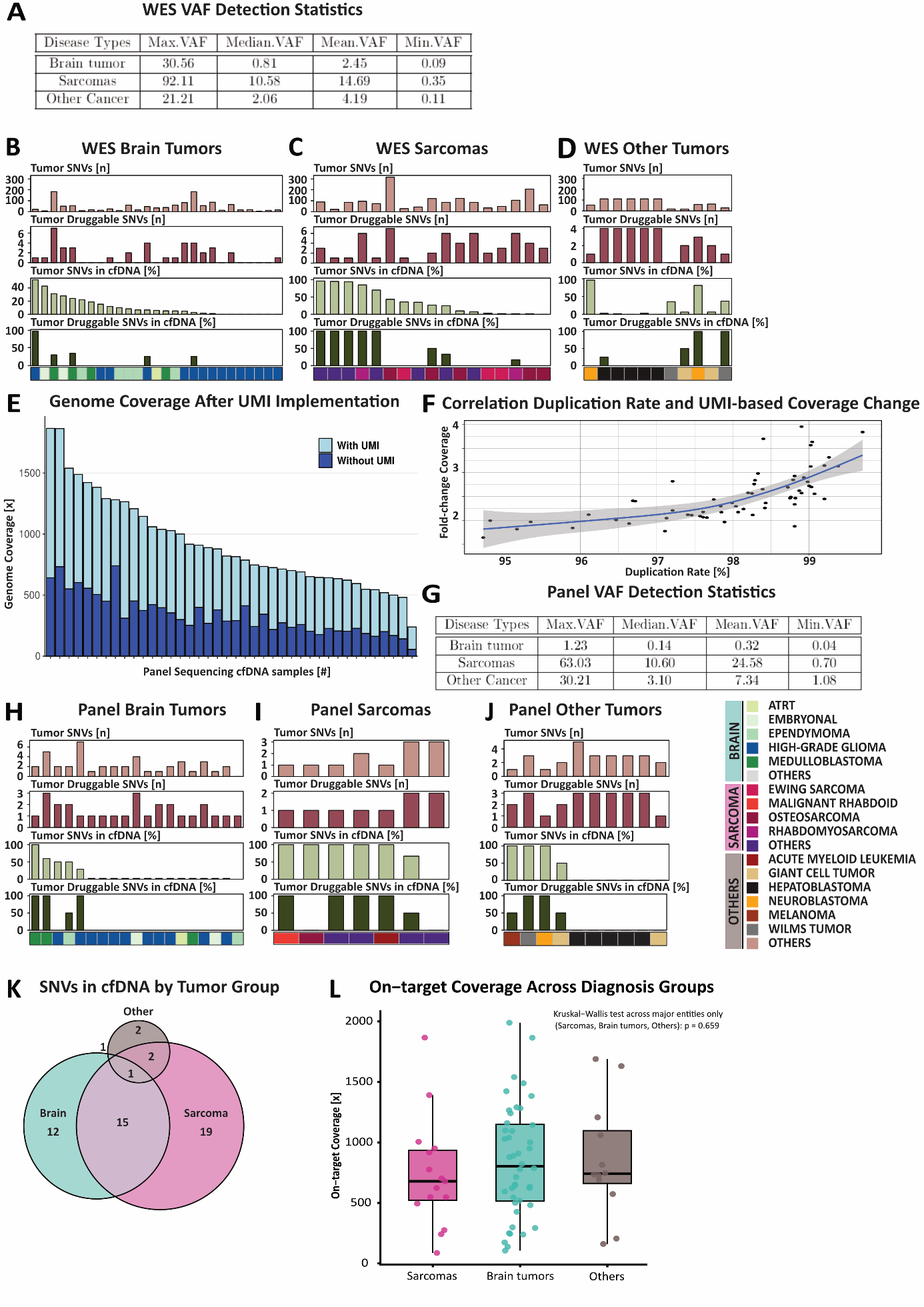


**Fig. S6 Sequencing Specifics and Single Nucleotide Variant (SNV) Detection Rate of Whole-Exome Sequencing (WES) and Targeted Panel Sequencing A)** Descriptive variant allele frequency (VAF, %) of tumor-informed SNVs detected in cell-free DNA (cfDNA) WES data. **B-D)** Bar plots representation for patient-specific absolute number of tumor SNVs (n), tumor druggable SNVs (n), relative detection of tumor SNVs in cfDNA (%), and relative detection of tumor druggable SNVs in cfDNA (%) WES data **B)** of individual brain tumor, **C)** sarcoma, and **D)** other tumor liquid biopsies. **E)** On-target mean depth of coverage of cfDNA panel sequencing data compared between regular MarkDuplication procedure and the UMI-based deduplication workflow. UMI-based deduplication (light blue) improved on-target mean depth of coverage by around 3-fold to regular MarkDuplication (dark blue). **F)** Increasing on-target mean depth of coverage in panel sequencing data correlated with high degree of duplicated reads removed by MarkDuplication procedure. **G)** Descriptive VAF (%) of tumor-informed SNVs detected in cfDNA panel sequencing data. **H-J)** Bar plots of panel sequencing data of individual brain tumor**, I)** sarcoma, and **J)** other tumor patients. **K)** Venn diagram showing the overlap of SNVs detected in cfDNA across tumor groups (brain tumors, sarcomas, and other entities). Numbers indicate the count of unique and shared variants between groups, highlighting both tumor-type–specific and overlapping mutational profiles. **L)** On-target panel sequencing coverage across diagnosis groups. Boxplots show the distribution of on-target sequencing coverage (×) across major pediatric diagnosis including sarcomas, brain tumors, and other tumor types. Center lines indicate the median, boxes represent the interquartile range (IQR), and whiskers extend to 1.5 × IQR. Individual points represent individual samples. Kruskal–Wallis test, p = 0.659.


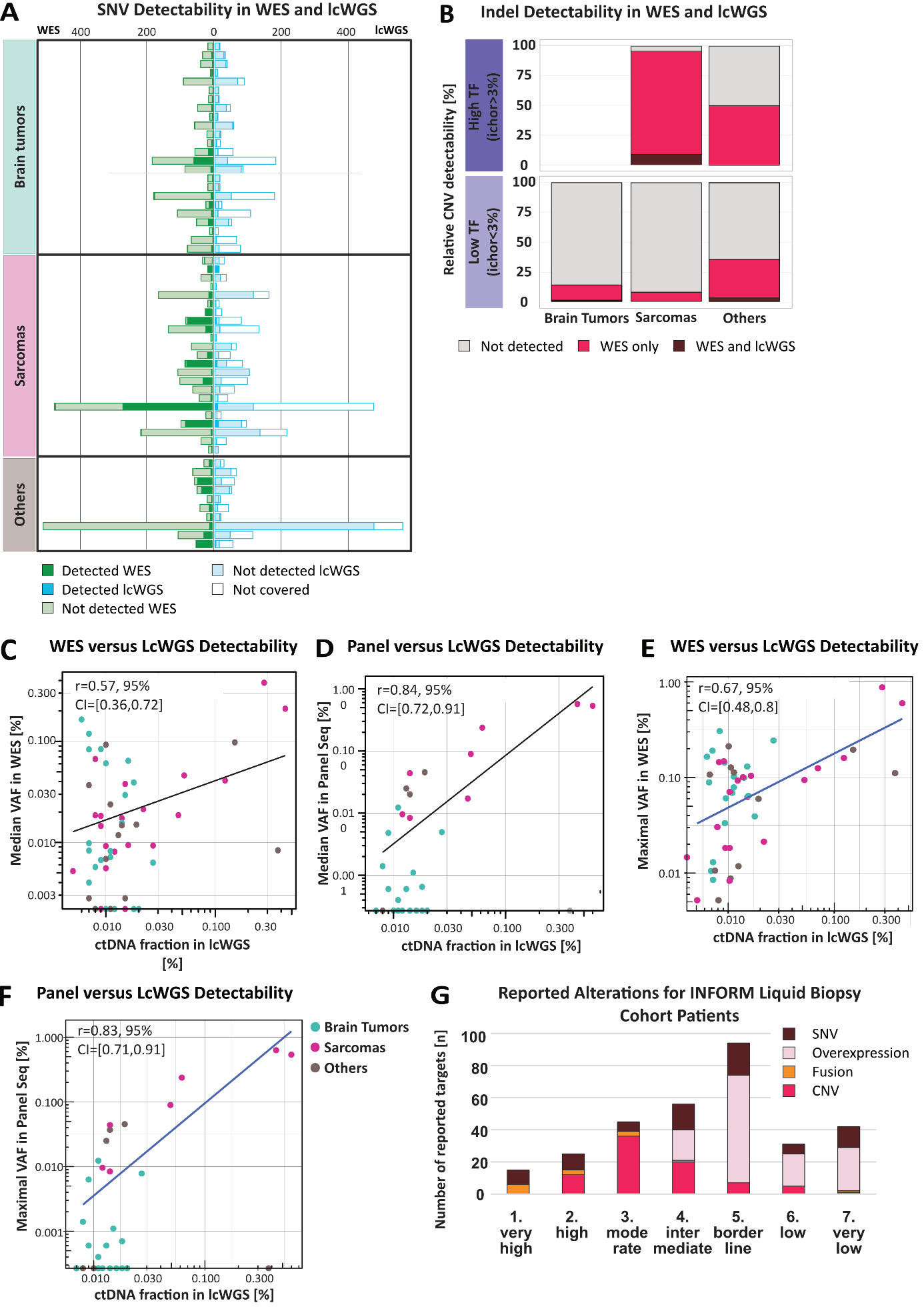


**Fig. S7** **Comparison of Orthogonal NGS Approaches for Detection of Tumor-derived Alterations in cell-free DNA (cfDNA) A)** Plasma-based detection of single nucleotide variants (SNVs) that were reported for matched tumors applying whole-exome (WES) and low-coverage whole-genome sequencing (lcWGS). WES: tumor SNVs detected (green) or not detected (light green) in cfDNA despite sufficient mean depth of coverage. LcWGS: tumor SNVs detected (blue) or not detected (light blue) in cfDNA despite sufficient mean depth of coverage. Tumor SNVs for which no sufficient mean depth of coverage was reached are indicated with white bars. **B)** Detection of small insertions and deletions (Indels) in cfDNA applying WES and lcWGS. Red – Indel detected by WES only, dark red – Indel detected by WES and lcWGS, grey – not detected by any method**. C-D)** Correlation plots of median variant allele frequencies (VAF) of detected tumor-informed mutations in **C)** WES and **D)** panel data with lcWGS-derived cell-free tumor DNA (ctDNA) fractions of matched cfDNA samples. **E-F)** Correlation of maximal VAF of detected tumor-informed mutations in **E)** WES and **F)** in panel data with lcWGS-derived ctDNA fractions of matched cfDNA samples. **G)** Overview of alteration types and target priority levels reported for corresponding tumors of patients in the liquid biopsy cohort within the INFORM molecular tumor board. Dark red – SNVs, light pink – overexpression, orange – fusions, red – copy-number variations (CNVs).
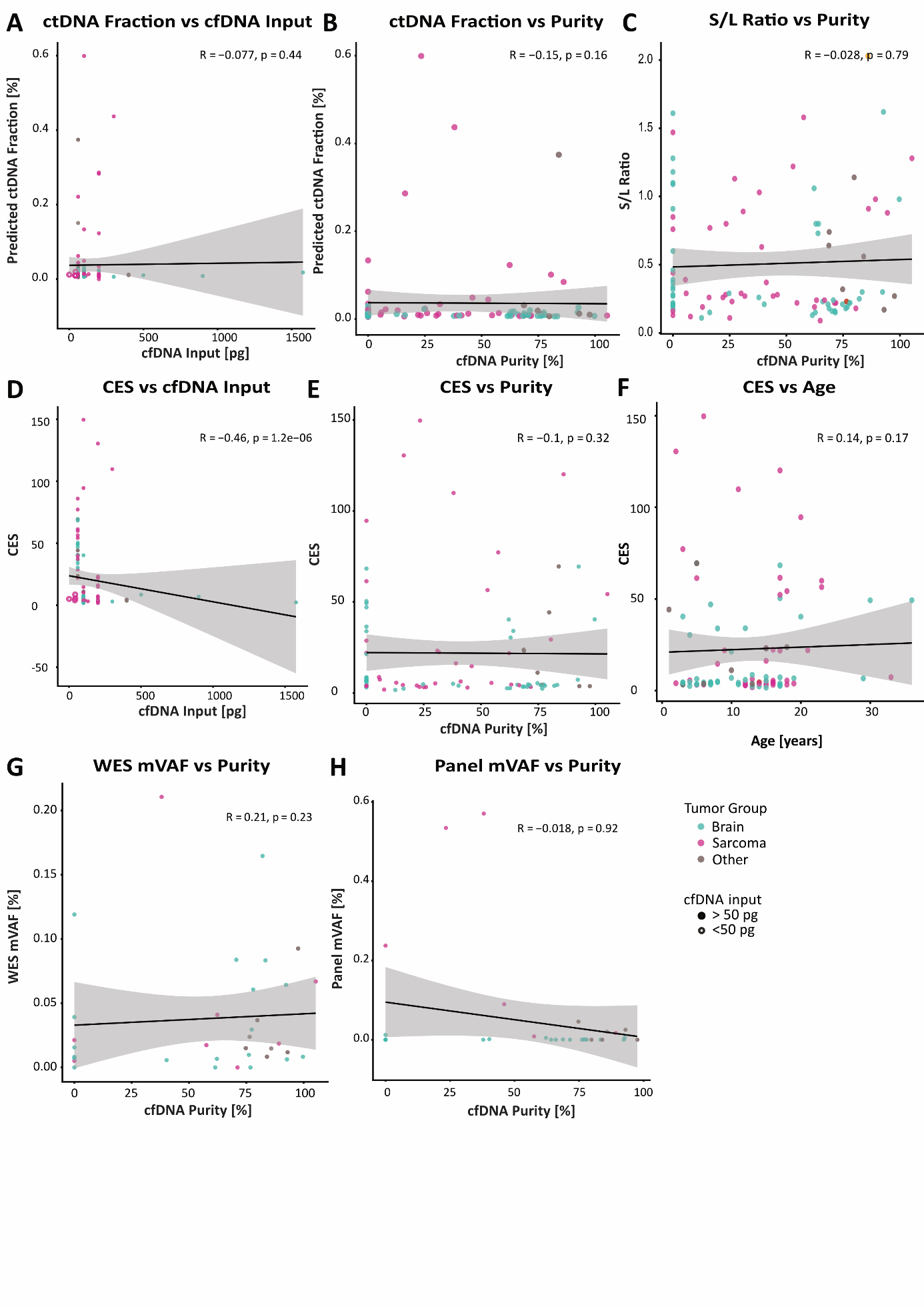


**Fig. S8 Relationship between cfDNA input, purity, and sequencing metrics A)** Correlation between cfDNA input (ng) and ctDNA fraction, with a weak negative association (Spearman R = −0.07, p = 0.55). Filled dots indicate samples with cfDNA input >50 pg, whereas open circles represent samples with cfDNA input ≤50 pg. **B)** Correlation between cfDNA purity (%) and ctDNA fraction showing no significant relationship (R = 0.05, p = 0.67). **C)** Correlation between cfDNA purity and S/L ratio (Spearman R = −0.028, p = 0.79). **D)** Correlation between cfDNA input and CES (R = −0.46, p = 1.2 × 10⁻^6^). **E)** Correlation between cfDNA purity and CES, with a weak negative association (Spearman R = −0.10, p = 0.32). **F)** Correlation between patients’ age and CES (Spearman R = 0.14, p = 0.17). **G)** Correlation between cfDNA purity and WES mean variant allele frequency (mVAF), showing a slight positive trend (Spearman R = 0.21, p = 0.07). **H)** Correlation between cfDNA purity and panel-derived mVAF, demonstrating a weak negative association (Spearman R = −0.15, p = 0.18). Each dot represents an individual sample. Samples are color-coded by tumor group. Solid lines indicate linear regression fits with shaded areas representing 95% confidence intervals.


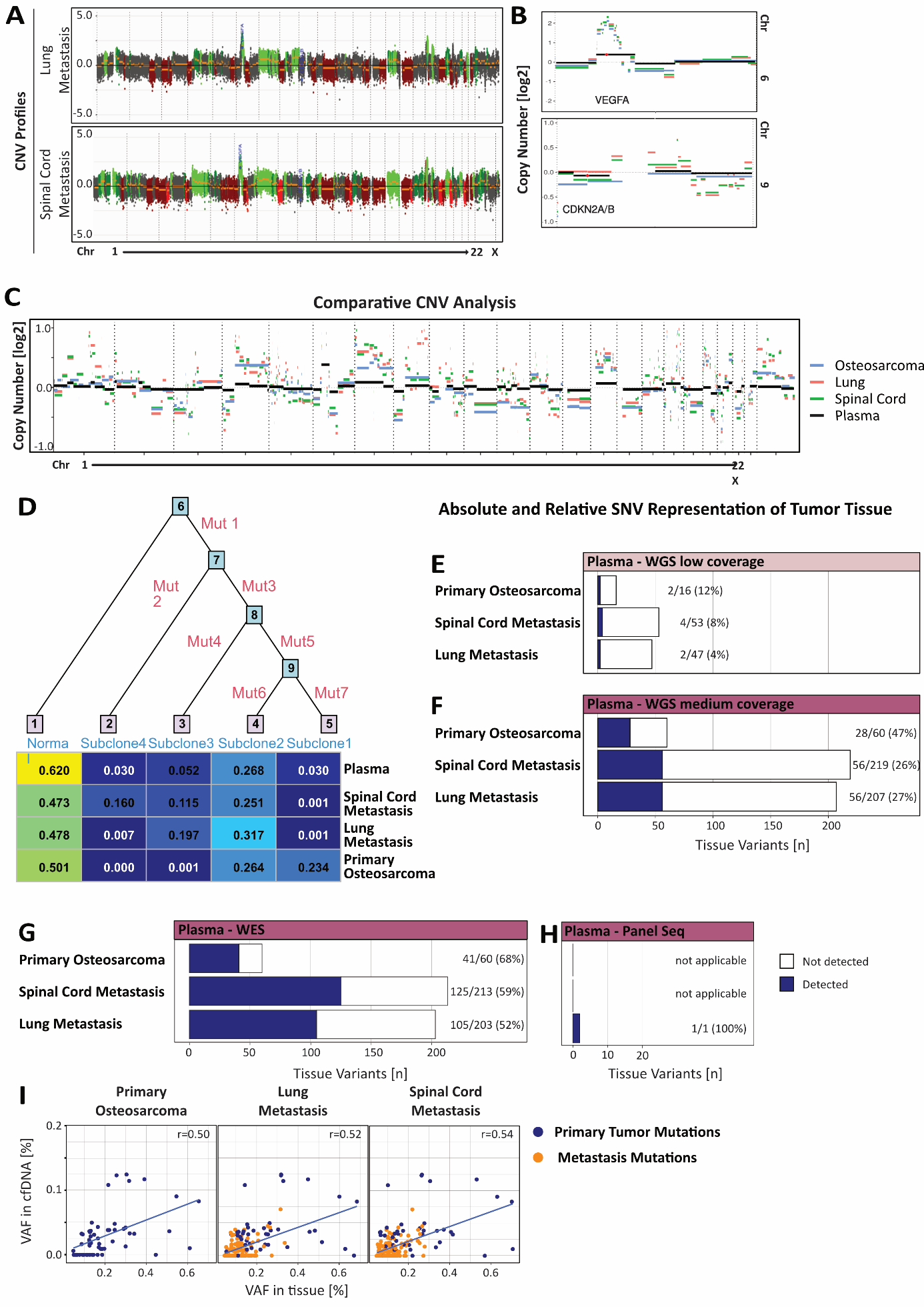


**Fig. S9 Liquid Biopsy-based Detection of Temporal Tumor Heterogeneity A)** Individual Copy Number Variation (CNV) profiles generated from whole-exome sequencing (WES) data from tumor tissue of lung and spinal cord metastases. **B)** Representative locus-specific CNV profiles for VEGFA (chr6) and CDKN2A/B (chr9) comparing different sample sites (the primary osteosarcoma (blue), a lung metastasis (41 months after diagnosis, green), a spinal cord metastasis (52 months after diagnosis, red) and plasma cfDNA (52 months after diagnosis, black). Log2 copy number ratios are shown, highlighting focal amplifications (*VEGFA*) and deletions (*CDKN2A/B*) detected across tumor sites and plasma. **C)** Comparative CNV analysis depicted as overlay plot of the primary osteosarcoma (blue), a lung metastasis (41 months after diagnosis, green), a spinal cord metastasis (52 months after diagnosis, red) and plasma cfDNA (52 months after diagnosis, black). **D)** Single nucleotide variant (SNV)-based phylogenetic trees depict variable clonal diversification. Branch lengths correspond to the total numbers of SNVs. Relative contribution of indicated subclones per tumor site and in the plasma sample. **E-H)** Absolute and relative detection rates of somatic tumor SNVs of primary osteosarcoma, spinal cord and lung metastases. **E-F)** Whole-genome sequencing (WGS) with **E)** low and **F)** medium mean depth of coverage revealed higher absolute numbers of somatic mutations in cell-free DNA (cfDNA) with medium mean depth of coverage, while relative numbers showed similar patterns. **G)** WES showed higher absolute and relative detectability of somatic SNVs than other sequencing methods. **H)** Panel sequencing only covered Notch1 mutation present in both the lung metastasis and cfDNA. **I)** Evolution of tumor SNVs and their respective variant allele frequencies (VAF) in tumor tissue (primary/metastasis, WES) and plasma-derived cfDNA (WES). Correlation plot of VAF in cfDNA and tissue of indicated tumor sites. Dark blue indicates SNVs detected in the primary osteosarcoma, orange indicates SNVs that evolved over time (r=0.50 (Osteosarcoma), r=0.52 (Lung Metastasis), r=0.54 (Spinal Cord Metastasis).


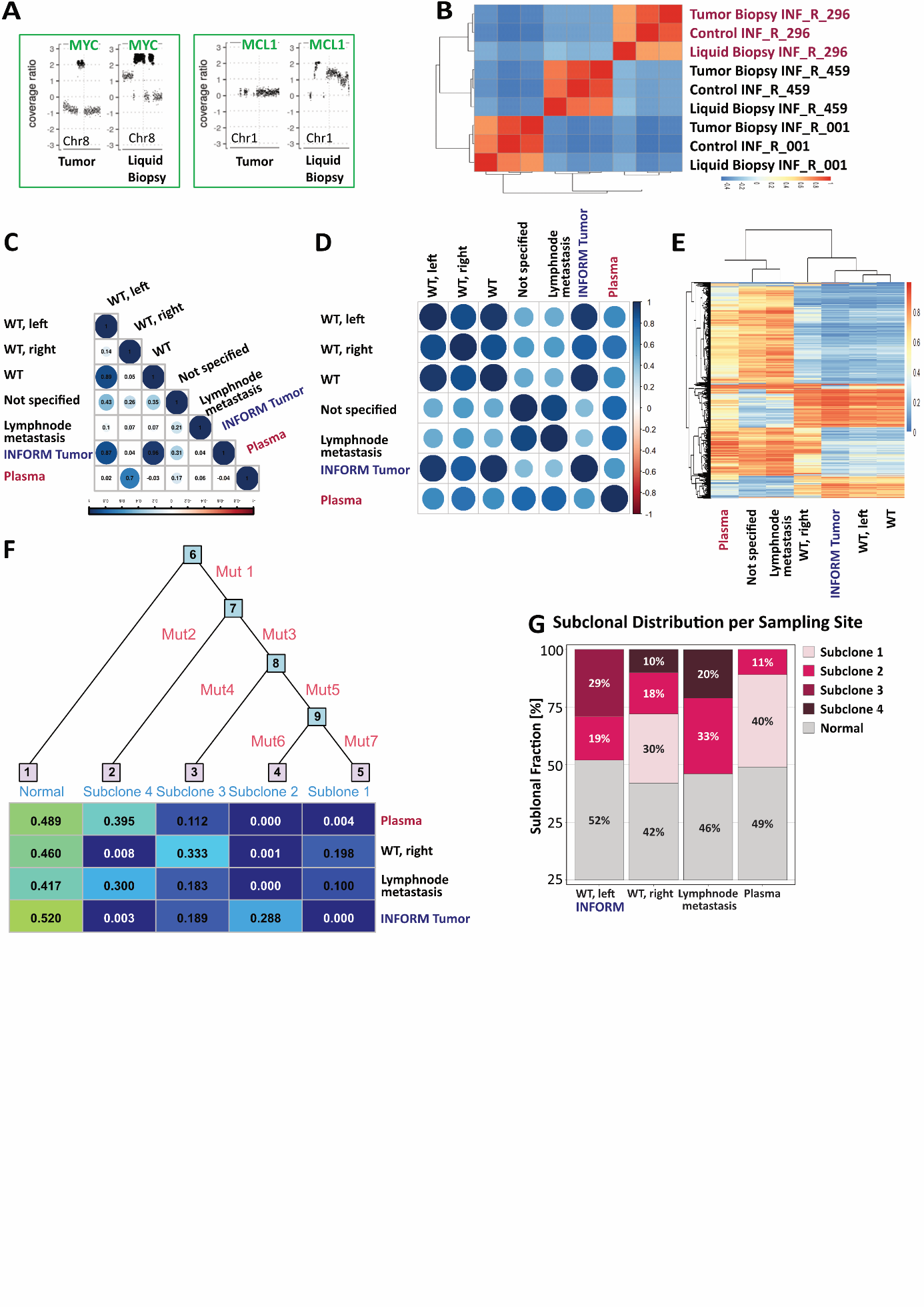


**Fig. S10 Liquid Biopsy-based Detection of Spatial Tumor Heterogeneity A)** MYC and MCL-1 amplifications as presented in the INFORM molecular tumor board here illustrated for tumor and liquid biopsy. **B)** Genotyping correlation matrix of 2 additional randomly selected patients including tumor biopsy, germline control and plasma cell-free DNA (cfDNA) each. The correlation coefficient suggests compatibility of all specimens from patient with Wilms tumor (WT). **C)** Pearson correlation of copy number variations (CNVs) for indicated tumor sites and plasma. Correlation coefficient indicated by color and circle size. **D)** Variance weighted Pearson correlation of methylation patterns from indicated biopsy samples revealed highest similarity between the plasma cfDNA clone and the Wilms tumor (WT) from the right site. **E)** Hierarchical clustering of the top 20,000 CpG probes with highest standard deviation indicated a methylation pattern unique to the WT of the right kidney and plasma clone. **F)** Single nucleotide variant (SNV)-based phylogenetic trees depict variable subclonal diversification. Branch lengths correspond to the total numbers of SNVs. **G)** Relative contribution of indicated subclones per tumor site and in the plasma sample
