# Supplementary material for: Circulating tumor DNA precision oncology enables effective and sensitive molecular diagnostics and actionable target detection in pediatric solid tumors - the INFORM experience
Source: Genome Med. 2026 Jul 27;18:112. doi: 10.1186/s13073-026-01737-4 (PMC13411563; doi:10.1186/s13073-026-01737-4)
Supplement: Supplementary file 2 — Additional file 2: Supplementary Methods [file 13073_2026_1737_MOESM2_ESM.docx]

# **Additional file 2**

## **cfdnakit SLRatio derivation and CES computation**

The SLRatio for each sample is defined as:

$$SLRatio=\frac{N_{short}}{N_{long}}$$

where $N_{short}$ is the total number of fragments of 100–150 nt

and $N_{long}$ is the total number of fragments of 151–250 nt genome-wide.

For bin-level analysis, read counts of short and long fragments per 1 Mb non-overlapping genomic window were each corrected for GC content and mappability bias using LOESS regression. The corrected per-bin SLRatio (${SL.Ratio}_{w}$) was then standardized within each sample by subtracting the sample median and dividing by the median absolute deviation (MAD) across all bins, excluding the bin under consideration:

$${SL.norm}_{w}=\frac{{SL.Ratio}_{w}-median\left( {SL.Ratio}_{1},\ldots, {SL.Ratio}_{n} \right)}{mad\left( {SL.Ratio}_{1},\ldots, {SL.Ratio}_{n} \right)}$$

where $w$ = ^44 𝑛^  and $n$ is the total number of bins.

The standardized per-bin value (${SL.norm}_{w}$) was then transformed into a z-score by normalizing against the distribution of the same bin across all PoN samples:

$${zscore}_{w}=\frac{{SL.norm}_{w}-median\left( {SL.norm}_{w,1},\ldots, {SL.norm}_{w,p} \right)}{mad\left( {SL.norm}_{w,1},\ldots, {SL.norm}_{w,p} \right)}$$

where $w$ = {1, 2, ..., $n$} and $n$ is the total number of bins

and $p$ = {1,2,… $m$}; where $m$ is number of samples in PoN

This two-step normalization first removes within-sample variability driven by the overall fragmentation profile, then removes bin-specific technical artifacts shared across the PoN, ensuring that residual signal reflects true tumor-associated fragmentation changes. Normalized bins were then segmented using circular binary segmentation (CBS), and the median z-score per segment ( $Z_{segment})$ was recorded alongside the number of bins in each segment ($l_{{segment}_{i}}$). These quantities feed directly into the CES formula described in the main Methods. Reads overlapping ENCODE DUKE and DAC blacklisted regions and centromeres were excluded from all fragment length analyses.

## **Comparison of internal and external healthy control cohorts**

To assess potential center-specific or protocol-specific effects on the healthy control reference distribution used for CES threshold derivation, we compared cfDNA biomarker metrics between two independent cohorts of healthy individuals: (1) internal healthy controls (n=10) and (2) a publicly available cfDNA dataset from healthy donors (EGA accession EGAD00001007080, n=22) sequenced at an independent center. CES scores, SLRatio values, and ichorCNA-estimated tumor fractions were compared between the two cohorts using the two-sided Mann-Whitney U test, with a significance threshold of α = 0.05. The absence of significant differences between cohorts supports the generalizability of the internally derived CES positivity threshold (CES = 3) across sequencing centers and sample processing protocols.
